# Effects of hormonal contraception on systemic metabolism: cross-sectional and longitudinal evidence

**DOI:** 10.1093/ije/dyw147

**Published:** 2016-08-18

**Authors:** Qin Wang, Peter Würtz, Kirsi Auro, Laure Morin-Papunen, Antti J Kangas, Pasi Soininen, Mika Tiainen, Tuulia Tynkkynen, Anni Joensuu, Aki S Havulinna, Kristiina Aalto, Marko Salmi, Stefan Blankenberg, Tanja Zeller, Jorma Viikari, Mika Kähönen, Terho Lehtimäki, Veikko Salomaa, Sirpa Jalkanen, Marjo-Riitta Järvelin, Markus Perola, Olli T Raitakari, Debbie A Lawlor, Johannes Kettunen, Mika Ala-Korpela

**Affiliations:** 1 Computational Medicine, Faculty of Medicine, University of Oulu & Biocenter Oulu, Oulu, Finland,; 2 NMR Metabolomics Laboratory, School of Pharmacy, University of Eastern Finland, Kuopio, Finland,; 3 National Institute for Health and Welfare, Helsinki, Finland,; 4 Institute for Molecular Medicine (FIMM), University of Helsinki, Helsinki, Finland,; 5 Department of Obstetrics and Gynecology, Helsinki University Central Hospital and University of Helsinki, Helsinki, Finland,; 6 Department of Obstetrics and Gynecology, Oulu University Hospital, University of Oulu and Medical Research Center Oulu, Oulu, Finland,; 7 Department of Medical Microbiology and Immunology, University of Turku, Turku, Finland,; 8 Clinic for General and Interventional Cardiology, University Heart Center Hamburg, Hamburg, Germany,; 9 German Center for Cardiovascular Research (DZHK e.V.), partner site Hamburg, Lübeck, Kiel, Germany,; 10 Department of Medicine, University of Turku and Division of Medicine, Turku University Hospital, Finland,; 11 Department of Clinical Physiology, University of Tampere and Tampere University Hospital, Tampere, Finland,; 12 Department of Clinical Chemistry, Fimlab Laboratories, School of Medicine, University of Tampere, Tampere, Finland,; 13 Department of Epidemiology and Biostatistics, MRC-PHE Centre for Environment and Health, School of Public Health, Imperial College London, UK,; 14 Institute of Health Sciences & Biocenter Oulu, University of Oulu, Oulu, Finland,; 15 Unit of Primary Care, Oulu University Hospital, Oulu, Finland,; 16 Estonian Genome Center, University of Tartu, Tartu, Estonia,; 17 Research Centre of Applied and Preventive Cardiovascular Medicine, University of Turku, Turku, Finland,; 18 Department of Clinical Physiology and Nuclear Medicine, Turku University Hospital, Turku, Finland,; 19 Medical Research Council Integrative Epidemiology Unit at the University of Bristol, Bristol, UK and; 20 School of Social and Community Medicine, University of Bristol, Bristol, UK

**Keywords:** hormonal contraception, combined oral contraceptive pills, progestin-only contraceptives, metabolomics, cytokines, inflammation, amino acids, fatty acids, lipoproteins, hormones, risk factors

## Abstract

**Background:** Hormonal contraception is commonly used worldwide, but its systemic effects across lipoprotein subclasses, fatty acids, circulating metabolites and cytokines remain poorly understood.

**Methods:** A comprehensive molecular profile (75 metabolic measures and 37 cytokines) was measured for up to 5841 women (age range 24–49 years) from three population-based cohorts. Women using combined oral contraceptive pills (COCPs) or progestin-only contraceptives (POCs) were compared with those who did not use hormonal contraception. Metabolomics profiles were reassessed for 869 women after 6 years to uncover the metabolic effects of starting, stopping and persistently using hormonal contraception.

**Results:** The comprehensive molecular profiling allowed multiple new findings on the metabolic associations with the use of COCPs. They were positively associated with lipoprotein subclasses, including all high-density lipoprotein (HDL) subclasses. The associations with fatty acids and amino acids were strong and variable in direction. COCP use was negatively associated with albumin and positively associated with creatinine and inflammatory markers, including glycoprotein acetyls and several growth factors and interleukins. Our findings also confirmed previous results e.g. for increased circulating triglycerides and HDL cholesterol. Starting COCPs caused similar metabolic changes to those observed cross-sectionally: the changes were maintained in consistent users and normalized in those who stopped using. In contrast, POCs were only weakly associated with metabolic and inflammatory markers. Results were consistent across all cohorts and for different COCP preparations and different types of POC delivery.

**Conclusions:** Use of COCPs causes widespread metabolic and inflammatory effects. However, persistent use does not appear to accumulate the effects over time and the metabolic perturbations are reversed upon discontinuation. POCs have little effect on systemic metabolism and inflammation.

**Key Messages**

- This work is the first comprehensive molecular characterization of the systemic effects of combined oral contraceptive pills (COCPs) and progestin-only contraceptives (POCs). Given the infeasibility of randomizing women to hormonal contraception or placebo, and the difficulty of randomizing to hormonal or non-hormonal contraception, evidence for causal effects requires well-conducted observational studies.
- The novel findings on the systemic effects of COCPs reported here are multiple. Use of COCPs increased the concentrations of circulating lipoprotein subclasses, including all high-density lipoprotein (HDL) subclasses. It resulted in changes in fatty acids and amino acids that were strong and variable in magnitude and decreased the circulating albumin levels but increased the concentrations of creatinine and multiple inflammatory markers, including glycoprotein acetyls and several growth factors and interleukins.
- We confirmed previous findings of increased circulating triglycerides, HDL cholesterol, apolipoprotein B and A-I, insulin, C-reactive protein, sex hormone-binding globulin and decreased testosterone in the COCP users.
- Most of the metabolic aberrations caused by the use of COCPs are associated with higher cardiometabolic risk based on established risk factors and also on the basis of multiple new metabolomics biomarkers.
- Persistent use of COCPs does not appear to accumulate the effects over time and the metabolic perturbations are reversed upon discontinuation. Use of POCs has little effect on systemic metabolism and inflammation.

## Introduction

Use of hormonal contraception is widespread: around 80% of women from high-income countries have used oral contraceptive pills.^1^^,^^2^ United Nations estimates the worldwide prevalence of hormonal contraception use among reproductive women in a union to be over 13%. Hormonal contraception offers convenient, effective and reversible fertility regulation, but the combined (oestrogen and progestin) oral contraceptive pills (COCPs) are associated with three to seven times higher risk of venous thrombosis and around a 2-fold risk of myocardial infraction and ischemic stroke.^3–7^ A large number of cross-sectional studies have shown that COCPs are associated with cardiometabolic risk markers, such as increased circulating levels of triglycerides and various cholesterol measures, and also greater insulin resistance and inflammatory marker C-reactive protein (CRP).^8–12^ This is in contrast to progestin-only contraceptives (POCs) that do not appear to be associated with higher venous thrombosis or other cardiometabolic risk.^3^^,^^4^^,^^6^^,^^7^^,^^10^

Randomized controlled trials have compared effects between different hormonal contraceptive preparations on metabolic risk markers.^13–16^ However, it is not feasible to compare the use of a hormonal contraceptive with non-use in a randomized trial, since few women would be prepared to be randomized to placebo or non-hormonal contraception. Longitudinal studies are therefore essential for determining the metabolic consequences of starting, stopping and continued use of hormonal contraception, and also for providing the best estimates of causal effects. However, most previous studies have been cross-sectional and only assessed a limited range of traditional metabolic risk factors.^8^ The few existing small longitudinal studies have suggested that the effects of COCPs on lipids and insulin resistance tend to appear shortly after starting the use, and that the effects do not worsen with continued use and that they reverse upon stopping.^8^^,^^14^^,^^17^^,^^18^ Our work here provides a plethora of new molecular information on the influences of hormonal contraception on a wide range of circulating markers of high relevance in cardiovascular risk assessment and in individual considerations on the decision of contraception.

## Methods

### Study populations

Data from three independent population-based Finnish cohorts were analysed: the 1997 survey of the Northern Finland Birth Cohort 1966 (NFBC1966, *n* = 2962 women aged 31 years),^19^ the 2001 survey of the Cardiovascular Risk in Young Finns Study (YFS, *n* = 1239 women aged 24–39 years)^20^ and the FINRISK 1997 study (*n* = 2105 women aged 24–49 years).^21^ Pregnant women (*n* = 337) and those whose information on contraceptive use was missing (*n* = 128) were excluded. In total, 5841 women who had the metabolomics profiles and information on contraceptive use were included in the study (Table 1). A subset of 869 women out of 1154 women at baseline in the YFS attended a 6-year follow-up at which contraceptive use was assessed and the metabolomics measurements repeated. The loss of 285 women at the follow-up is due to no attendance at the clinical examination (*n* = 201), no data on metabolic profiles (*n* = 36), no information on contraception use (*n* = 18) or pregnancy at the follow-up (*n* = 30). The study protocols were approved by the local ethics committees and all participants gave written informed consents. Further details of the study populations^19–21^ are given in Supplementary Methods (supplementary data are available at *IJE* online).

**Table 1.** Characteristics of study participants

Characteristics	NFBC1966			YFS			FINRISK1997		
	Control	COCP	POC	Control	COCP	POC	Control	COCP	POC
Number of individuals	1915	585	188	727	298	129	1507	274	218
Percentage of users (%)*	65	20	6	59	24	10	72	13	10
Age (year)	31.1 (0.4)	31.2 (0.4)	31.1 (0.4)	32.1 (4.9)	29.5 (4.8)	34.5 (4.0)	38.3 (6.9)	31.6 (5.4)	37.4 (6.4)
BMI (kg/m^2^)	24.3 (4.8)	23.4 (3.7)	24.4 (4.6)	24.7 (4.8)	23.7 (3.9)	25.0 (5.1)	25.2 (4.7)	23.2 (3.6)	24.7 (4.1)
Systolic blood pressure (mmHg)	119 (12)	122 (12)	119 (13)	112 (12)	115 (13)	112 (13)	125 (15)	122 (12)	123 (16)
Diastolic blood pressure (mmHg)	75 (10)	76 (11)	74 (11)	69 (10)	70 (10)	69 (10)	78 (10)	75 (10)	77 (11)
Smoking prevalence (%)	37	34	47	20	21	21	23	24	25
Alcohol usage (g/day)	2.1 (0.5, 5.7)	3.3 (1.1, 7.3)	2.7 (1.1, 6.2)	3.3 (0.0, 8.2)	4.9 (1.6, 9.9)	3.3 (0.0, 8.2)	1.8 (0.0, 7.0)	3.5 (0.0, 8.0)	2.1 (0.0, 8.3)
Plasma glucose (mmol/L)	4.9 (0.5)	4.9 (0.4)	4.9 (0.5)	4.9 (0.6)	4.9 (0.5)	5.1 (1.5)	4.9 (0.8)	4.8 (0.9)	4.8 (0.6)
Insulin (IU/L)	7.2 (5.9, 9.0)	8.0 (6.5, 9.6)	7.0 (5.7, 8.6)	6 (4, 9)	7 (5, 10)	6 (4, 9)	4.5 (3.2, 6.5)	5.1 (4.0, 7.4)	4.7 (3.3, 6.5)
HDL cholesterol (mmol/L)	1.7 (0.4)	2.0 (0.5)	1.6 (0.5)	1.7 (0.4)	1.9 (0.4)	1.6 (0.3)	1.7 (0.3)	1.9 (0.4)	1.7 (0.3)
Total cholesterol (mmol/L)	5.1 (1.1)	5.5 (1.2)	4.9 (1.1)	4.9 (1.0)	5.2 (1.0)	4.8 (0.9)	5.0 (0.9)	5.1 (1.0)	4.9 (0.9)
Triglycerides (mmol/L)	0.8 (0.6, 1.1)	1.1 (0.9, 1.4)	0.8 (0.6, 1.1)	1.0 (0.8, 1.3)	1.2 (0.9, 1.5)	0.9 (0.7, 1.2)	0.9 (0.7, 1.2)	0.9 (0.7, 1.1)	0.9 (0.6, 1.2)

Values are mean (standard deviation) for normally distributed and median (interquartile range) for skewed variables. COCP, combined oral contraceptive pill; POC, progestin-only contraceptive; BMI, body mass index; HDL, high-density lipoprotein. *Percentage of users is defined as the percentage of contraceptive users among all the women who had a metabolomics profile measured. The characteristics of the subgroups of COCP and POC users are given in Supplementary Table 1 (available as at *IJE* online).

### Information on hormonal contraception use and covariates

Use of contraception, smoking status and alcohol consumption were assessed by questionnaires. Body mass index (BMI) and blood pressure were assessed in clinics using established protocols.

Information on the oestrogen dose and the type of progestin for COCP preparations was available for NFBC1966 and YFS2001. None of the cohorts had information on how long women had used hormonal contraception.

We undertook two primary analyses: (i) comparing women using COCPs with those using no hormonal contraception and (ii) comparing women using any form of POC—including pills, implants and intrauterine systems (IUSs)—with those using no hormonal contraception. Thus, women were categorized into three mutually exclusive groups: (i) non-users of any hormonal contraception (*n* = 4149; including women using no contraception and those using non-hormonal means, such as barrier methods and non-hormonal intrauterine devices), (ii) users of COCPs (*n* = 1157) and (iii) users of POCs (*n* = 535). In secondary analyses, we compared (i) different generations of COCPs with non-users of hormonal contraception and (ii) different forms of POC delivery with non-users. Thus, COCPs were categorized into second-generation (oestrogen and levonorgestrel/norgestimate) and third-generation (oestrogen and desogestrel/gestodene) pills and those containing oestrogen and cyproterone acetate. No participants reported the use of other preparations of COCPs e.g. drospirenone. The POCs were further categorized into progestin-only pills and levonorgestrel-IUS.

The vast majority of women who were using a COCP used a preparation with either 20 mcg or 30–40 mcg of ethinylestradiol. The hormonal contraception methods analysed here represent the common preparations with respect to progestin type and ethinylestradiol dosage in widespread use.^3^^,22^ Characteristics of the study participants in different contraceptive groups are given in Supplementary Table 1 (available as Supplementary data at *IJE* online).

### Molecular profiling

Seventy-five metabolic measures were assessed, with 68 of these quantified by a high-throughput serum nuclear magnetic resonance (NMR) metabolomics platform.^23^^,^^24^ These measures represent a broad molecular signature of systemic metabolism and cover multiple metabolic pathways, including lipoprotein lipids and subclasses, fatty acids, amino acids and glycolysis-related metabolites. The NMR-based metabolomics profiling has previously been used in large-scale epidemiological studies^25–31^ and the experimentation described elsewhere.^23^^,^^24^^,^^32^ Six hormone-related measures (insulin, leptin, adiponectin, vitamin D, sex hormone-binding globulin and testosterone), high-sensitivity CRP and 37 cytokines were also analysed. Details of these measurements are given in Supplementary Methods and Supplementary Appendix 1 (available as Supplementary data at *IJE* online).

### Statistical analyses

The metabolic and cytokine measures were log-transformed and scaled to standard deviations (SD) in each cohort. A multiple testing corrected threshold *P* < 0.0004 (0.05/112 measures) was used to indicate statistical significance.

For cross-sectional analyses, a linear regression model was fitted for each outcome measure (concentration of each molecular measure) with the contraception group as the explanatory variable. Non-users of hormonal contraception were used as the reference group, so that association magnitudes denote the difference in each outcome measure between hormonal contraceptive users and non-users. Association magnitudes are reported in SD units throughout in order to ease the comparison across multiple measures. The three cohorts were analysed separately and the results then combined via an inverse variance weighted meta-analysis using fixed effects model, after confirming the consistency of the metabolic associations across the three cohorts (Supplementary Figure 1, available as Supplementary data at *IJE* online). In the main analyses, we adjusted for potential confounding by age, which is related to the type of contraception used^33^ and affects lipid and metabolite levels.^34^ In a second set of models, we additionally adjusted for BMI, mean arterial pressure [MAP, calculated as 1/3 × (systolic blood pressure) + 2/3 × (diastolic blood pressure)], current smoking and alcohol consumption, which could potentially also confound the associations.^8–10^

Those 869 women from the YFS cohort who had both baseline and 6-year follow-up data were classified as starters, stoppers and persistent users of COCP (*n* = 235); starters, stoppers and persistent users of IUS (*n* = 176); switchers of IUS and COCP (*n* = 34); and persistent non-users (*n* = 392). The other contraception users (in total 32 women) were excluded in the longitudinal analyses due to their small number. The contingency table of the contraceptive users at baseline and follow-up is given in Supplementary Table 4 (available as Supplementary data at *IJE* online). For each metabolic measure, the 6-year change in concentration for starters, stoppers and persistent users of COCPs or IUSs and for the switchers were compared with those for persistent non-users. The longitudinal models were adjusted for baseline age and further for the 6-year change of BMI, MAP, smoking and alcohol use.

## Results

The characteristics of the study participants are shown in Table 1. On average, 19% and 9% of women were using COCPs and POCs, respectively. Characteristics of POC users were broadly similar to those of non-users, whereas COCP users tended to be younger, leaner and consumed more alcohol than the other two groups. Blood pressure and smoking levels were similar in all three groups.

### Metabolic profiles of COCP and POC use

**Figure 1.**
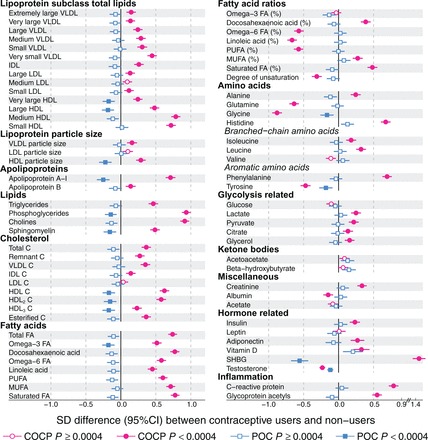
Cross-sectional associations of the use of combined oral contraceptive pills (COCPs) and progestin-only contraceptives (POCs) with 75 molecular measures. Non-users of any hormonal contraception were used as the reference group, so the association magnitudes denote the difference in each measure between hormonal contraceptive users and non-users. Association magnitudes are reported in standard deviation-units to ease the comparison across multiple measures. Associations were adjusted for age and meta-analysed for three independent population-based cohorts. In total, 1157 women using COCPs and 535 using POCs were compared with 4149 non-users of hormonal contraception. VLDL, very-low-density lipoprotein; IDL, intermediate-density lipoprotein; LDL, low-density lipoprotein; HDL, high-density lipoprotein; C, cholesterol; FA, fatty acids; PUFA, polyunsaturated fatty acids; MUFA, monounsaturated fatty acids; SHBG, sex hormone-binding globulin. Open and closed symbols indicate *P* ≥ 0.0004 and *P* < 0.0004, respectively.

The cross-sectional associations of COCP and POC use with 75 metabolic measures are shown in Figure 1

(results in absolute physiological units are given in Supplementary Table 2, available as Supplementary data at *IJE* online). Use of COCPs was strongly associated with almost the entire molecular profile (65 out of 75 measures at *P* < 0.0004 in the meta-analysis). The concentrations of total lipids in all lipoprotein subclasses were increased; the strongest associations were for the high-density lipoprotein (HDL) subclasses and for the smallest very-low-density lipoprotein (VLDL) subclasses. Only slight increases were observed for low-density lipoprotein (LDL) subclasses. Concomitantly, apolipoprotein A-I, apolipoprotein B, triglyceride and various cholesterol concentrations, except LDL cholesterol, increased. Likewise, the concentrations of all circulating fatty acids were markedly elevated. However, the proportion of individual fatty acids (relative to the total fatty acid concentration) displayed a heterogeneous association pattern. The proportions of omega-6 fatty acids, including linoleic acid, were markedly decreased whereas the proportion of docosahexaenoic acid as well as monounsaturated and saturated fatty acids were increased.

The use of COCPs was also associated with large differences in amino acid concentrations, with histidine and phenylalanine increasing the most, whereas glutamine, glycine and tyrosine decreased substantially. Glycolysis-related metabolites were moderately associated with the use of COCPs whereas the ketone bodies were not. COCP use was also associated with increased serum concentrations of creatinine, insulin, adiponectin, vitamin D and sex hormone-binding globulin (SHBG) and decreased concentrations of albumin and testosterone. In addition, COCP use was strongly linked with increased levels of inflammatory markers, CRP and glycoprotein acetyls. In contrast to the use of COCPs, the use of POCs were only weakly, or not at all, associated with the metabolic measures (Figure 1).

The cross-sectional results were highly consistent across the three independent cohorts (Supplementary Figures 1 and 2, available as Supplementary data at *IJE* online). Results were also very similar when further adjusted for BMI, MAP, current smoking and alcohol use (Supplementary Figure 3, available as Supplementary data at *IJE* online). All COCP subtypes/generations consistently showed strong associations with the molecular profile (Supplementary Figure 4, available as Supplementary data at *IJE* online) but POC subtypes, progestin-only pills and IUSs were only weakly associated (Supplementary Figure 5, available as Supplementary data at *IJE* online). Details are given in Supplementary Table 1 and Supplementary Appendix 2 (available as Supplementary data at *IJE* online).

The cross-sectional associations with 37 cytokines revealed that COCP use was associated with multiple inflammatory pathways, including angiogenesis- and hemotopoiesis-related growth factors as well as interleukins. POCs were only weakly associated with cytokines. The results are shown in Supplementary Figure 6, given in absolute concentration units in Supplementary Table 3 and detailed information provided in Supplementary Appendix 1 (all available as Supplementary data at *IJE* online).

### Metabolic responses of starting, stopping and persistently using COCPs

Figure 2

**Figure 2.**
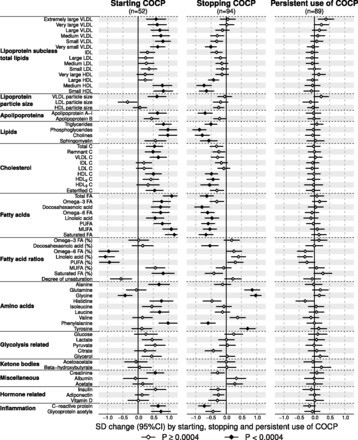
Longitudinal changes in molecular concentrations in response to starting, stopping and persistent use of combined oral contraceptive pills (COCPs). The 6-year metabolic changes for starting (*n* = 52), stopping (*n* = 94) and persistently using (*n* = 89) COCPs were compared with those of persistent non-users (*n* = 392) in the Young Finns Study (YFS) cohort. A null result for persistent users indicates metabolic changes consistent with those for the persistent non-users (i.e. changes that would occur with age or any secular event over the 6-years of follow-up) that is no further worsening effects were detected due to persistent use of COCPs. The marked changes for starters and stoppers, and their opposite directions, suggest that the metabolic effects were produced by starting to use COCPs and normalized by stopping the use. The longitudinal associations were adjusted for baseline age. Open and closed diamonds indicate *P* ≥ 0.0004 and *P* < 0.0004, respectively. Abbreviations are as for Figure 1.

illustrates metabolic changes in response to starting, stopping and persistently using COCPs. During the 6-year follow-up, there were only very small metabolic changes for the persistent users of COCPs in comparison with those women who were persistent non-users. For those women who started to use COCPs, there were pronounced metabolic changes across the entire molecular profile; the association magnitudes were highly similar to those observed in the cross-sectional setting. The metabolic changes were also pronounced for the women who stopped using COCPs; the association magnitudes again matched the cross-sectional findings, but they were in the opposite direction. The overall consistency between the longitudinal metabolic associations of starting the use of COCPs and the corresponding cross-sectional associations of using COCPs followed a straight line with a slope of 0.99 ± 0.08 (*R*^2 ^= 0.69; Figure 3

**Figure 3.**
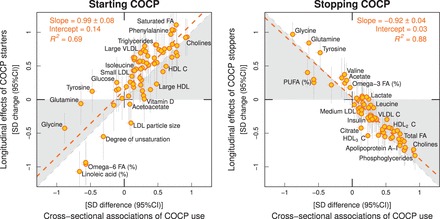
Correlation between cross-sectional and longitudinal metabolic associations with the use of combined oral contraceptive pills (COCPs). The correspondence of cross-sectional associations with starting and stopping the use of COCPs is shown on the left and right panels, respectively. Each point represents a single metabolic measure. Horizontal and vertical grey lines denote 95% confidence intervals for the cross-sectional and longitudinal associations, respectively. The grey shaded areas serve to guide the eye for the slope. A linear fit of the overall correspondence summarize the match between cross-sectional and longitudinal associations, with *R*^2^ denoting the goodness of fit. A slope of ±1 and *R*^2 ^= 1 would strongly support the causal effects of COCP use on the metabolic measures. Abbreviations are as for Figure 1.

, left panel). Analogously, the association magnitudes for those who stopped using COCPs also followed a straight line with downwards slope of –0.92 ± 0.04 (*R*^2 ^= 0.88; Figure 3, right panel). The metabolic associations for starting, stopping and persistently using COCPs were essentially unaltered when further adjusted for 6-year change of BMI, MAP, smoking and alcohol use (Supplementary Figure 7, available as Supplementary data at *IJE* online).

In contrast to the large metabolic perturbations related to starting and stopping the use of COCPs, there were essentially no metabolic changes associated with starting, stopping or persistently using IUSs (Supplementary Figure 8, available as Supplementary data at *IJE* online). The metabolic changes for those women who started or stopped using IUSs matched poorly with the cross-sectional associations with IUS use (*R*^2 ^< 0.3) (Supplementary Figure 9, available as Supplementary data at *IJE* online). Metabolic changes in response to the switch between COCPs and IUSs during the follow-up (Supplementary Table 4) are shown in Supplementary Figure 10 and discussed in Supplementary Appendix 3 (all available as Supplementary data at *IJE* online).

## Discussion

This study elucidates the widespread changes in systemic metabolism arising from the use of COCPs in unprecedented molecular detail. The metabolic effects of starting COCPs extend markedly beyond the small set of established cardiovascular risk factors assessed in previous studies.^8^^,^^14^^,^^17^^,^^18^ The comprehensive molecular profiling allowed multiple new findings of the wide systemic perturbations associated with the use of COCPs. In cross-sectional analyses, the use of COCPs was primarily associated with metabolic differences towards higher cardiometabolic risk, including substantial effects on numerous novel and emerging biomarkers for the risk of cardiovascular disease (CVD) and type 2 diabetes.^25^^,^^28^^,^^30^^,^^35^^,^^36^ The metabolic effects were pronounced, with magnitudes often around and even larger than 0.5 SD different from the non-users. Longitudinal analyses of starting and stopping the use of COCPs, together with the very large association magnitudes, strongly suggest that the metabolic aberrations arise as the cause of COCPs. Importantly, long-term use of COCPs does, however, not appear to have any accumulative metabolic effects. In contrast to the widespread effects of COCPs, any delivery method of POCs was only weakly, or not at all, associated with the molecular profile. The null associations of the IUS use in the longitudinal settings reinforce that IUSs, and potentially other POCs, are unlikely to cause marked systemic metabolic deviations.

The molecular underpinnings of COCP use have been widely studied with respect to glucose metabolism and routine lipids. Numerous observational studies and randomized trials have found increased triglycerides, apolipoprotein B, apolipoprotein A-I and insulin to be associated with COCP use.^11^^,^^14^^,^^17^^,^^18^^,^^37–40^ These measures, together with the inflammatory marker CRP, circulating testosterone and SHBG, serve here as positive controls, providing evidence of validity for results with our much more detailed profiling in the same cohorts. The lipoprotein subclass data revealed that the use of COCPs increased circulating lipids in all VLDL subclasses, particularly the smaller ones. This, together with increased intermediate-density lipoprotein (IDL) as well as higher levels of triglycerides and remnant cholesterol, indicate higher CVD risk for the COCP users. Recent evidence from Mendelian randomization analyses suggest that higher levels of triglyceride-rich lipoproteins and remnant cholesterol cause CVD.^41–43^ Over recent decades, changes to COCP formulas have aimed at maximizing their HDL-cholesterol-elevating properties whilst minimizing other risks. Previous studies have reported that users of second-generation pills had no change or decreased HDL cholesterol, whereas users of third- or newer-generation pills displayed increased HDL cholesterol.^11^^,^^14^^,^^17^^,^^18^^,^^37–39^ Here, the HDL subclass data revealed that both second- and third/newer-generation pills increased medium and small HDL subclasses in a similar manner, but only the third/newer-generation products resulted in robust increases in larger HDL subclasses. The concomitant increases in the smaller HDL subclasses for the second- and third-generation pills are in line with previous studies.^11^^,^^37^ Although third- and newer-generation COCPs result in higher levels of HDL cholesterol than the second generation, mostly due to increases in the larger HDL subclasses, these newer preparations displayed 50–80% higher risk of venous thrombosis than the second generation, and have previously been shown to convey similar risk of myocardial infraction and ischemic stroke to older preparations.^5^^,^^6^^,^^22^ The lack of difference in ischemic heart disease when comparing newer- to older-generation COCPs, despite higher HDL cholesterol levels, is consistent with recent randomized controlled trials and Mendelian randomization studies that suggest that HDL cholesterol is not causally protective of CVD.^42^^,^^44^^,^^45^ In fact, recent findings suggest that high circulating HDL cholesterol may also be related to an increased risk for CVD.^46^

The metabolomics profiling allowed an overall characterization of the circulating fatty acids. The use of COCPs increased circulating lipoprotein lipids, which is also reflected in the increased fatty acid concentrations. However, the relation between COCP use and the fatty acid balance revealed a mixed set of associations, with mostly adverse aberrations in terms of CVD risk.^30^ The use of COCPs was adversely associated with decreased proportion of omega-6 fatty acids and increased proportions of monounsaturated and saturated fatty acids.^30^^,^^36^ Nevertheless, increased proportion of docosahexaenoic acid, an omega-3 fatty acid, suggests a favourable link between the use of COCPs and the risk for CVD.^30^ The potential causal role of these circulating fatty acids in the CVD pathogenesis remains elusive.^30^^,^^47^

Recent metabolic profiling studies have linked multiple circulating amino acids and other small molecules with the risk of CVD, type 2 diabetes and all-cause mortality.^25^^,^^28^^,^^30^^,^^48^ Use of COCPs appears to perturb multiple amino acid pathways. The association pattern of COCP use with branched-chain and aromatic amino acids seems unique, as it appears not to follow the consistent elevations previously seen with obesity and insulin resistance.^25^^,^^29^ There have been small studies (fewer than 30 COCP users) assessing the metabolic associations of COCP use with amino acids.^49–53^ Consistently with our findings, these much smaller studies have generally found COCP use associated with lower levels of glycine and tyrosine. However, the previous results for other amino acids appear to be inconsistent. Our cross-sectional and longitudinal analyses both suggest that the use of COCPs results in increased phenylalanine and decreased tyrosine levels. The opposite direction of effect is surprising given their intrinsically positively linked metabolism. However, these findings appear robust, since the association magnitudes are notable and consistent across three independent cohorts. This divergent pattern of increased phenylalanine and decreased tyrosine has been seen previously in patients with chronic kidney diseases.^54^^,^^55^ As the kidneys are the chief source of circulating tyrosine in a fasting state,^56^ a possible impairment of phenylalanine conversion to tyrosine in the kidneys has been suggested.^54^^,^^55^ The current observation that COCP use is associated with increased creatinine concentrations is supported by a trial in which all subtypes of COCPs were associated with higher levels of creatinine.^57^ In Finnish women of similar age, higher serum creatinine in the COCP users was thus associated with lower estimated glomerular filtration rate^58^—a marker of decreased kidney function and higher risk of CVD mortality.^59^ We also found that the use of COCPs was adversely associated with circulating albumin—a marker that has previously been linked to the risk of diabetic renal diseases, CVD and all-cause mortality.^28^^,^^48^ Overall, some of the metabolic changes in response to the use of COCPs, including increased phenylalanine and decreased tyrosine, higher creatinine and lower albumin, resemble metabolic characteristics of impaired kidney function. In addition, the increased concentrations of multiple inflammatory markers, including CRP and glycoprotein acetyls, point towards a possibility of elevated inflammation in the COCP users (more detailed discussion given in Supplementary Appendix 1, available as Supplementary data at *IJE* online). Mendelian randomization analyses have argued against the adverse causal role of higher CRP for CVD^60^^,^^61^ and challenged the cardio-protective role of vitamin D.^62^^,^^63^ However, the potential causal role of other biomarkers remains currently unclear.

Studies that have reported increased CVD risk in COCP users have mostly studied women at reproductive age, during which the absolute risk for CVD events is generally low e.g. approximately two incidences of arterial thrombosis and seven incidences of venous thrombosis per 10 000 person-years in current users of third-generation COCPs.^3^^,^^6^ Given the already evident causal role of multiple metabolic measures shown here to be adversely affected by COCP, a life-time accumulation of CVD risk is anticipated.^64^ Although it is reassuring to see that the metabolic effects of long-term COCP use are normalized upon stopping, it is currently unclear how much temporary disruptions in circulating CVD risk factors can affect the lifelong risk for CVD. Due to the widespread use of COCPs, often for decades, further studies are needed to evaluate the potential effects of accumulative exposure of COCP use at reproductive age on the subsequent CVD risk at older age.

None of the POC methods (pills or IUSs) was robustly associated with metabolic perturbations. These findings suggest that ethinylestradiol (the most common oestrogen used in COCPs),^9^ alone or in interaction with progestin, is largely responsible for the broad metabolic effects of COCP use. This is supported by the recent findings that COCPs with higher dose of ethinylestradiol are associated with higher risk of venous thrombosis, thrombotic stroke and myocardial infarction.^5^^,^^6^ Furthermore, administration of ethinylestradiol has been shown to affect various circulating lipid levels, hepatic proteins and clotting factors.^15^^,^^65^

The strengths of this study include extensive molecular profiling of systemic metabolism with replication across three large population-based cohorts. This is thereby the first comprehensive molecular characterization of the effects of COCP use. Longitudinal metabolomics data in relation to starting and stopping the use of COCPs provided strong evidence that the use of COCPs is the direct cause of the systemic metabolic changes. We were able to adjust for a wide range of relevant confounders, but we acknowledge that we were not able to adjust for confounding by indications for why a woman uses contraception or a particular type of contraception. However, we anticipate the metabolic effects of such indications to be very small in comparison to the strong perturbations observed.

Given the infeasibility of randomizing women to use hormonal contraception or placebo, and the difficulty of randomizing to hormonal or non-hormonal contraception, evidence for causal effects in this field requires well-conducted observational studies. Also, using Mendelian randomization analysis to assess the causal effects of oestrogen and progestin is hampered, since identification of genetic variants for oestrogen and progesterone has to date proved elusive.^66^^,^^67^ Furthermore, if such variants were identified, they might not mimic the effects of taking exogenous hormones. The presented longitudinal study setting is therefore the best realistically available way to infer causality.

In conclusion, our comprehensive profiling of molecular markers of systemic metabolism showed that starting the use of COCPs perturbs multiple metabolic pathways with pronounced changes predominantly associated with higher cardiometabolic risk. Persistent use of COCPs did not appear to accumulate metabolic risk over a 6-year period and the discontinuation normalized the metabolic profile. The use of POCs had only minor effects on the circulating metabolic profile, suggesting that the effects of the COCP use are largely due to ethinylestradiol. Whilst we recognize that a wide range of considerations are important when contemplating whether and what type of contraception to use, and that pregnancy itself has adverse health consequences,^68^ we feel that understanding the extensive metabolic effects of hormonal contraception is one of the key aspects that women and their health-care providers should be aware when making these decisions. Our findings also provide extra means to optimize the type of contraception on the basis of the individual risk profile.

## Funding

The quantitative serum NMR metabolomics platform and its development have been supported by the Academy of Finland, TEKES—the Finnish Funding Agency for Technology and Innovation, Sigrid Juselius Foundation, Novo Nordisk Foundation, Finnish Diabetes Research Foundation, Paavo Nurmi Foundation and strategic and infrastructural research funding from the University of Oulu, Finland, as well as by the British Heart Foundation, the Welcome Trust and the UK Medical Research Council. The Young Finns Study has been financially supported by the Academy of Finland: grant numbers 134309 (Eye), 126925, 121584, 124282, 129378 (Salve), 117797 (Gendi), 41071 (Skidi), and 286284, the Social Insurance Institution of Finland, Kuopio, Tampere and Turku University Hospital Medical Funds, Juho Vainio Foundation, Sigrid Juselius Foundation, Yrjö Jahnsson Foundation, Paavo Nurmi Foundation, Finnish Foundation for Cardiovascular Research and Finnish Cultural Foundation, Tampere Tuberculosis Foundation and Emil Aaltonen Foundation. MS and SJ have been supported through funds from the Academy of Finland (grant number 141136). NFBC1966 has received financial support from the Academy of Finland (project grants 104781, 120315, 129269, 1114194 and SALVE), University Hospital Oulu, Biocenter, University of Oulu, Finland (75617), European Commission (EURO-BLCS, Framework 5 award QLG1-CT-2000–01643; EU H2020-PHC-2014-grant no. 633595) and the UK Medical Research Council (G0500539, G0600705, PrevMetSyn/SALVE, PS0476) and the Wellcome Trust (project grant GR069224). KA has received funding from Finnish Medical Association. SB and TZ have been supported by European–Union Seventh Framework Programme (FP7/2007–2013) under grant agreement No. HEALTH-F2‐2011–278913 (BiomarCaRE). VS has received funds from the Academy of Finland (grant number 139635) and from Finnish Foundation for Cardiovascular Research. JK was supported through funds from the Academy of Finland (grant number 283045). DAL and MA-K work in a Unit that receives funds from the University of Bristol and the UK Medical Research Council (MC_UU_12013/1, MC_UU_12013/5). DAL is a UK National Institute of Health Senior Investigator (NF-SI-0611–10196). The funders had no role in study design, data collection and analysis, decision to publish or preparation of the manuscript.

*Conflict of interest:* PW, AJK, PS and MAK are shareholders of Brainshake Ltd (www.brainshake.fi), a company offering NMR-based metabolic profiling. QW, PW, AJK, PS, MT, TT and JK report employment and consulting for Brainshake Ltd. SB has received research funding from Boehringer Ingelheim, Bayer, Abbott Diagnostics, SIEMENS, Thermo Fisher and Roche Diagnostics and received honoraria for lectures or consulting from Boehringer Ingelheim, Bayer, Roche, Astra Zeneca, SIEMENS, ThermoFisher and Abbott Diagnostics. DAL has received funds from Medtronic PLC, Roche Diagnostics and Ferring Pharmaceuticals for biomarker research that is unrelated to the work in this paper. Other authors did not report any disclosures.

## Supplementary Material

Supplementary Data
