# Supplementary material for: Effects of hormonal contraception on systemic metabolism: cross-sectional and longitudinal evidence
Source: Int J Epidemiol. 2016 Aug 18;45(5):1445–57. doi: 10.1093/ije/dyw147 (PMC5100613; doi:10.1093/ije/dyw147)
Supplement: Supplementary Data [file dyw147_supplementary_data.zip › ije-2015-09-1236-File005.pdf]

Supplementary Material

Wang Q, Würtz P, Auro K, et al. Effects of hormonal contraception on systemic metabolism: cross-sectional and longitudinal evidence.

Supplementary Methods

Study populations.

Supplementary Appendixes

Appendix S1. Associations of hormonal contraception with cytokines.

Appendix S2. Associations of COCP and POC subtypes with metabolic measures.

Appendix S3. Effects of switching the contraceptive method on metabolic measures.

Supplementary Tables

Table S1. Characteristics of the study participants in different contraceptive groups.

Table S2. Molecular differences associated with the use of combined oral contraceptive pills and progestin-only contraceptives in absolute concentration units.

Table S3. Cytokine differences associated with the use of combined oral contraceptive pills and progestin-only contraceptives in absolute concentration units.

Table S4. Contingency table of contraceptive users at baseline and follow-up in YFS.

Supplementary Figures

Figure S1. Cross-sectional associations between the use of combined oral contraceptive pills and 75 molecular measures in the individual cohorts.

Figure S2. Cross-sectional associations between the use of progestin-only contraceptives and 75 molecular measures in the individual cohorts.

Figure S3. Cross-sectional associations of the use of combined oral contraceptive pills and progestin-only contraceptives with 75 molecular measures, adjusted for age, BMI, mean arterial pressure, smoking and alcohol use.

Figure S4. Cross-sectional associations between the use of different subtypes of combined oral contraceptive pills with 75 molecular measures.

Figure S5. Cross-sectional associations between the use of different subtypes of progestin-only contraceptives with 75 molecular measures.

Figure S6. Cross-sectional associations of the use of combined oral contraceptive pills and progestin-only contraceptives with 37 cytokines.

Figure S7. Longitudinal changes in molecular concentrations in response to starting, stopping and persistent use of combined oral contraceptive pills, adjusted for baseline age, and 6-y changes in

BMI, mean arterial pressure, smoking and alcohol use.

Figure S8. Longitudinal changes in molecular concentrations in response to starting, stopping and persistent use of intrauterine systems.

Figure S9. Correlation between cross-sectional and longitudinal metabolic associations with the use of intrauterine systems.

Figure S10. Longitudinal changes in molecular concentrations in response to the change of the contraceptive type.

Supplementary Methods

Study populations

The Northern Finland Birth Cohort of 1966 (NFBC1966) was initiated to study factors affecting preterm birth and subsequent morbidity in the two northernmost provinces in Finland. It included 12 058 children born alive, comprising 96% of all births during 1966 in the region.[1] Data collection in 1997 included clinical examination and serum sampling at the age of 31 years for 6007 individuals. Data from this time point are analyzed in the present study. Attendees in the field study at age 31 (70%) were representative of the original cohort.[1] Metabolomics were measured for 2962 women, of which 96% were based on fasting serum samples.[2] Pregnant women (n=202) and those who had missing information on the contraception (n=72) were excluded. In total, 2688 women who had the metabolomics profile measured and information on contraception were included in this work.

Contraception use, current smoking status and average alcohol usage were assessed from questionnaires. Blood pressure was measured using a mercury sphygmomanometer. Plasma insulin, vitamin D, and high-sensitivity C-reactive protein (CRP) were measured by standard clinical chemistry assays. Testosterone was measured by mass spectrometry. Sex hormone-binding globulin (SHBG) was not measured for the women using hormonal contraception and therefore this measure was not used in the analysis from this cohort. Informed written consent was obtained from all participants, and the research protocols were approved by the Ethics Committee of Northern Ostrobothnia Hospital District, Finland.

The Cardiovascular Risk in Young Finns Study (YFS) was designed to study associations of childhood risk factors to cardiovascular disease in adulthood.[3] The baseline study conducted in 1980 included 3596 children and adolescents aged 3–18 years. Data from the 2001 survey included 2247 individuals with an overnight fasting metabolomics profile measured (response rate 63%). These individuals were representative of the baseline cohort.[3] Data from this time-point are used in this study. Pregnant women (n=61) and those who had missing information on the contraception (n=24) were excluded.

In total, 1154 women who had the metabolomics profile measured and information on contraception available were included for the present analyses. A subset of 869 women who attended a follow-up assessment had metabolomics data and information on contraception also 6 years later. These 869 women had similar clinical characteristics as the ones who were not available for the follow-up analysis. In addition, 1101 women from the follow-up had cytokine panels measured (see Appendix S1).

Contraception use, current smoking status and average alcohol usage were assessed by questionnaires. Blood pressure was measured using a random-zero sphygmomanometer. The following biomarkers were measured by standard clinical chemistry assays and analyzed in the present study: insulin, leptin, adiponectin, vitamin D, and high-sensitivity CRP. SHBG was measured by Spectria SHBG IRMA and testosterone was measured by Spectria Testosterone kit. CRP and all the mentioned hormone-related measures were analyzed for the baseline. All but leptin, SHBG and testosterone were measured at the 6-year follow-up. All participants gave written informed consent, and the study was approved by the ethics committees of each five participating medical university study sites.

At the follow-up, women were asked if they use oral contraceptive pills, with no means of distinguishing combined oral contraceptive pills (COCPs) from progestin-only pills. However, the baseline data shows that the vast majority of women on oral contraception were using COCPs (92% across all three studies and 97% in the YFS baseline; Table S1 and S4). In addition, there were no clear associations of progestin-only contraceptives with metabolic measures at baseline (Figure1, main paper), making it appropriate to presume that the influences of oral contraceptive pills would be almost entirely from COCPs. Thus, all oral contraceptive users were treated as COCP users at the follow-up.

The FINRISK 1997 Study (FINRISK) was conducted to monitor the health of the Finnish population among persons aged 24–74 at recruitment.[4] In total, 8444 individuals were recruited to represent the middle-aged population of the study areas. Metabolomics from serum samples were measured for 3829 women. The median fasting time was 5h (interquartile range 4–6h). Women aged over 49 (n=1724), pregnant (n=74), and those with missing information on contraception use (n=32) were excluded. In total, data on serum metabolomics, cytokines (Appendix S1), and contraception use were available for 1999 women.

Contraception use, current smoking status and average use of alcohol were assessed by questionnaires. Blood pressure was measured using a mercury sphygmomanometer. The following circulating biomarkers were assayed by standard clinical chemistry assays and analyzed in the present study: insulin, leptin, adiponectin, vitamin D, testosterone, and high-sensitivity CRP. Participants gave written informed consent and the FINRISK study was approved by the Coordinating Ethical Committee of the Helsinki and Uusimaa Hospital District.

Supplementary Appendixes

Appendix S1. Associations of hormonal contraception with cytokines.

Cytokine profiling

YFS: Total of 48 cytokines were measured for 2200 individuals in the 2007 follow-up survey using Bio-Rad's premixed Bio-Plex Pro Human Cytokine 27-plex Assay and 21-plex Assay, and Bio-Plex 200 reader with Bio-Plex 6.0 software.[5] The assays were performed according to manufacturer's instructions, except, that the amount of beads, detection antibodies and streptavidin-phycoerythrin conjugate were used with 50% lower concentrations than recommended by the manufacturer. Only measures within the cytokine-specific detection range were included in the analyses. Low absolute concentrations of several cytokines (with respect to the sensitivity of the method) complicate their quantification.

The Bio-Rad analyser program fitted the measured light signals from the individual samples to the standard curves generated with recombinant cytokines for each cytokine on each 96-well plate using a five-parameter logistic regression. Due to the non-linear standard curves, the upper and lower detection limits are calculated plate-wise, so that they corresponded with "asymptotic" concentrations representing fluorescent intensity 2% above lower and 2% below upper asymptote of the calibration curve. If more than 50% of the observations corresponded to the asymptotic concentrations or were missing (i.e., below the detection limit) for a particular measure, it was excluded from further analyses. A similar exclusion criterion has been used in a recent publication using the same data.[5] This type of quality control resulted in 11 cytokine measures to be excluded; 37 measures were subsequently used in the further analyses.

FINRISK: The same Bio-Plex assays were used to quantify the cytokines as in YFS. Eighteen of the 37 measures that passed the quality control in YFS were also available in FINRISK.

In total, 3100 women had the cytokine panel measured (n=1101 from YFS follow-up and n=1999 from FINRISK).

Cytokine profiles of COCP and POC use

The associations of COCP and POC use with circulating cytokines are shown in Figure S6. The corresponding results in absolute physiological units are listed in Table S3. The use of COCPs was strongly associated with several cytokine measures. Prominent positive associations were observed for most of the growth factors, with the largest differences observed for a hematopoietic growth factor – stem cell growth factor beta ($SCGF_{\beta}$). Strong positive associations were also observed for beta nerve growth factor (βNGF), stem cell factor (SCF) and angiogenesis related markers including vascular endothelial growth factor (VEGF), basic fibroblast growth factor (FGF basic), and platelet derived growth factor BB (PDGF-BB). Chemokines were generally only weakly associated with COCP use except monokine induced by interferon-gamma (MIG), which showed a strong positive association. Interleukin-2 receptor alpha (IL-2rα), interleukin-12p70 (IL-12p70) and interleukin-17 (IL-17) were also markedly increased. However, some widely studied cytokines such as interleukin-1-beta (IL-1β), interleukin-6 (IL-6) and tumor necrosis factor-alpha (TNFα) displayed weak and non-significant associations. Tumor necrosis factor-related apoptosis inducing ligand (TRAIL) was inversely associated with the use of COCPs.

In contrast to the use of COCPs, the use of POCs was only weakly associated with the cytokines (Figure S6). For those cytokine measures that were available both in YFS and FINRISK, the results were largely consistent between the cohorts. The associations were very similar when further adjusted for BMI, MAP, current smoking and alcohol use.

Summary

Use of COCPs is associated with increased levels of inflammatory markers, including CRP and glycoprotein acetyls (GlycA). A randomised controlled study of 35 women has reported that COCP use is positively associated with CRP and shows no associations with IL-6 or TNF-alpha.[6] Therefore, it has been suggested that increased CRP concentration during COCP use might result from a direct effect on hepatocyte synthesis of CRP and may not reflect general inflammation. Emerging evidence elsewhere has also argued against CRP as a universal marker of general inflammation. On the other hand, elevated GlycA, a new inflammatory biomarker which reflects the circulating levels of various inflammatory glycoproteins, and is associated with both acute-phase and chronic inflammation, has been suggested as a likely direct consequence of increased neutrophil activities.[5] Ritchie et al. also reported that in apparently healthy individuals GlycA is broadly associated with both anti- and pro-inflammatory cytokines but the association magnitudes are generally modest/weak, suggesting a low-grade inflammation in the individuals with high GlycA. In the current work, we noticed that COCP use was robustly associated with higher levels of GlycA (≥0.5 SD), while the positive associations of COCP use with IL-1 beta, IL-6 and TNF alpha (comparing between up to 495 COCP users and 2186 non-users) tended to be rather weak (≤ 0.2 SD) with large confidence intervals. Large studies would therefore be needed to be able to reliably conclude whether COCP use is related to inflammation and/or hepatic acute phase protein release.

Appendix S2. Associations of COCP and POC subtypes with metabolic measures.

COCPs are commonly used by women in reproductive age worldwide and they constituted 70% of the hormonal contraception in this population-based study from Finland. COCPs mostly contain ethinylestradiol and progestin, and they are typically classified according to the type of progestin used.[7] In secondary analyses, we compared i) different generations of COCPs with non-users of hormonal contraception and ii) different forms of POC delivery with non-users. Thus, women using COCPs were further categorized into the following subgroups: 1) users of second generation COCPs, containing estrogen and levonorgestrel/norgestimate (n=189), 2) users of third generation COCPs, containing estrogen and desogestrel/gestodene (n=600), and 3) users of COCPs containing estrogen and cyproterone acetate (CPA, n=138). Information on the second and third generation COCPs was available for NFBC1966 and YFS2001. Information on the CPA pills was available for all the three cohorts (Table S1). Women using POCs were further categorized into the following subgroups: 1) users of progestin-only pills (n=94), and 2) users of intrauterine systems (IUSs) (n=434, all with levonorgestrel-IUS). Users of implants were excluded due to their small number (n=7).

Among the COCP subtypes, the third generation pills were the most common (used by about 15% of all study participants), followed by the second generation pills (4%), and the CPA pills (3%). The use of COCPs was associated to a broadly similar extent with the molecular measures for all subtypes (Figure S4). In general, the use of third generation and CPA pills was associated

with larger metabolic differences than the use of second generation pills, with the most pronounced differences observed for various HDL-related measures, phosphoglycerides, cholines, fatty acids, phenylalanine, insulin, SHBG, testosterone, and GlycA. These findings are in line with the 50-80% higher risk for venous thromboembolism linked with third generation and CPA pills compared to second generation pills.[7] The use of both progestin-only pills and IUSs was only weakly, or not at all, associated with the molecular profile (Figure S5).

Appendix S3. Effects of switching the contraceptive method on metabolic measures.

Metabolic changes in response to the switch between COCPs and IUSs during the follow-up are shown in Figure S10. The swap from the use of COCPs to using an IUS was associated with extensive metabolic changes corresponding to the pattern seen for those women who stopped using COCPs (Figure 2, main paper). For those women who swapped from using an IUS to using COCPs, a metabolic association pattern similar to those who started using COCPs (Figure 2, main paper) was noticeable despite data were available for only five women.

References

1. Järvelin M-R, Sovio U, King V, Lauren L, Xu B, McCarthy MI, et al. Early life factors and blood pressure at age 31 years in the 1966 northern Finland birth cohort. Hypertension 2004;44:838–46.
2. Würtz P, Mäkinen V-P, Soininen P, Kangas AJ, Tukiainen T, Kettunen J, et al. Metabolic signatures of insulin resistance in 7,098 young adults. Diabetes 2012;61:1372–80.
3. Raitakari OT, Juonala M, Rönnemaa T, Keltikangas-Järvinen L, Räsänen L, Pietikäinen M, et al. Cohort profile: the cardiovascular risk in Young Finns Study. Int J Epidemiol 2008;37:1220–6.
4. Blankenberg S, Zeller T, Saarela O, Havulinna AS, Kee F, Tunstall-Pedoe H, et al. Contribution of 30 biomarkers to 10-year cardiovascular risk estimation in 2 population cohorts: the MONICA, risk, genetics, archiving, and monograph (MORGAM) biomarker project. Circulation 2010;121:2388–97.
5. Ritchie SC, Würtz P, Nath AP, Abraham G, Havulinna AS, Fearnley LG, et al. The Biomarker GlycA Is Associated with Chronic Inflammation and Predicts Long-Term Risk of Severe Infection. Cell Systems 2015;1:293–301.
6. van Rooijen M, Hansson LO, Frostegard J, Silveira A, Hamsten A, Bremme K. Treatment with combined oral contraceptives induces a rise in serum C-reactive protein in the absence of a general inflammatory response. J Thromb Haemost 2006;4:77–82.
7. Stegeman BH, de Bastos M, Rosendaal FR, van Hylckama Vlieg A, Helmerhorst FM, Stijnen T, et al. Different combined oral contraceptives and the risk of venous thrombosis: systematic review and network meta-analysis. BMJ 2013;347:f5298.
8. Soininen P, Kangas AJ, Würtz P, Suna T, Ala-Korpela M. Quantitative serum nuclear magnetic resonance metabolomics in cardiovascular epidemiology and genetics. Circ Cardiovasc Genet 2015;8:192–206.

Table S1. Characteristics of the study participants in different contraceptive groups.

Characteristics	Control	Combined oral contraceptive pills			Progestin-only contraceptives		
		Second generation	Third generation	CPA	Progestin-only pills	IUS	Implant*
**NFBC1966**							
Number of individuals	1915	149	391	45	20	164	4
Percentage of users[%][†]	65	5	13	2	1	6	0
Age [year]	31.1 (0.4)	31.2 (0.4)	31.1 (0.4)	31.1 (0.3)	31.2 (0.4)	31.1 (0.4)	–
BMI [kg/m^2]	24.3 (4.8)	23.1 (3.1)	23.6 (3.9)	23.0 (3.4)	23.9 (4.4)	24.5 (4.7)	–
Systolic blood pressure [mmHg]	119 (12)	120 (10)	123 (13)	122 (11)	119 (11)	119 (13)	–
Diastolic blood pressure [mmHg]	75 (11)	76 (10)	76 (12)	78 (12)	74 (10)	74 (11)	–
Smoking prevalence [%]	37	35	34	30	56	45	–
Alcohol usage [g/day]	2.1 [0.5,5.7]	2.7 [1.1,6.7]	3.5 [1.1,7.5]	3.9 [0.9,6.9]	3.8 [2.5,8.3]	2.7 [1.1,5.8]	–
**YFS**							
Number of individuals	727	40	209	49	9	117	3
Percentage of users [%][†]	59	3	17	4	1	9	0
Age [year]	32.1 (4.9)	31.5 (5.0)	28.7 (4.2)	31.4 (5.9)	33.7 (4.9)	34.6 (3.9)	–
BMI [kg/m^2]	24.7 (4.8)	23.9 (5.2)	23.6 (3.7)	24.2 (3.6)	25.4 (5.6)	25.0 (5.1)	–
Systolic blood pressure [mmHg]	112 (12)	112 (14)	116 (13)	113 (11)	119 (20)	112 (12)	–
Diastolic blood pressure [mmHg]	69 (10)	69 (11)	71 (10)	69 (11)	73 (15)	68 (10)	–
Smoking prevalence [%]	20	30	22	11	33	19	–
Alcohol usage [g/day]	3.3 [0.0,8.2]	2.5 [0.0,11.5]	4.9 [1.6,9.9]	4.9 [3.3,9.9]	1.6 [0.0,8.2]	3.3 [0.0,8.2]	–
**FINRISK[‡]**							
Number of individuals	1507	–	–	44	65	153	0
Percentage of users [%][†]	72	–	–	2	3	7	0
Age [year]	38.3 (6.9)	–	–	34.5 (6.2)	33.4 (5.6)	39.1 (5.9)	–
BMI [kg/m^2]	25.2 (4.7)	–	–	23.4 (3.7)	23.8 (2.9)	25.1 (4.5)	–
Systolic blood pressure [mmHg]	125 (15)	–	–	121 (12)	121 (13)	124 (17)	–
Diastolic blood pressure [mmHg]	78 (11)	–	–	77 (9)	76 (10)	77 (11)	–
Smoking prevalence [%]	23	–	–	16	23	25	–
Alcohol usage [g/day]	1.8 [0.0,7.0]	–	–	1.9 [0.0,5.2]	1.8 [0.0,6.9]	2.1 [0.0,8.7]	–

Values are mean (SD) for normally distributed and median [interquartile range] for skewed variables. *Since there are less than 5 implant users in each cohorts, the characteristic were not reported for them. [†]percentage of users is defined as the percentage of contraceptive users among all the women who had metabolomics profiles. [‡]In FINRISK, there is no information to distinguish the second generation and third generation COCPs.

Table S2. Molecular differences associated with the use of combined oral contraceptive pills and progestin-only contraceptives in absolute concentration units.

Molecular measures	Mean (SD)	Absolute difference associated with COCP use in the cross-sectional setting<br><br>Beta [95%CI]; P-value	Absolute difference associated with POC use in the cross-sectional setting<br><br>Beta [95%CI]; P-value
**Lipoprotein subclass total lipids**			
Extremely large VLDL (µmol/L)	13 (18)	3.1 [1.9,4.3]; P=2e-06	-1.4 [-3.1,0.2]; P=0.06
Very large VLDL (µmol/L)	35 (45)	6.8 [3.9,9.8]; P=9e-06	-3.9 [-7.9,0.2]; P=0.05
Large VLDL (µmol/L)	150 (160)	37 [27,48]; P=4e-15	-13 [-27,0]; P=0.07
Medium VLDL (mmol/L)	0.4 (0.2)	0.06 [0.04,0.08]; P=4e-19	-0.01 [-0.03,0.01]; P=0.4
Small VLDL (mmol/L)	0.5 (0.2)	0.06 [0.04,0.07]; P=6e-21	-0.006 [-0.024,0.013]; P=0.8
Very small VLDL (mmol/L)	0.5 (0.2)	0.07 [0.06,0.08]; P=9e-46	-0.02 [-0.03,0.00]; P=0.05
IDL (mmol/L)	1.2 (0.3)	0.08 [0.06,0.10]; P=4e-16	-0.03 [-0.06,0.00]; P=0.04
Large LDL (mmol/L)	1.4 (0.4)	0.05 [0.02,0.08]; P=3e-05	-0.02 [-0.06,0.01]; P=0.2
Medium LDL (mmol/L)	0.8 (0.2)	0.02 [0.01,0.04]; P=0.004	-0.01 [-0.03,0.01]; P=0.3
Small LDL (mmol/L)	0.5 (0.2)	0.02 [0.01,0.03]; P=0.0003	-0.01 [-0.03,0.00]; P=0.1
Very large HDL (mmol/L)	0.6 (0.2)	0.07 [0.05,0.08]; P=1e-15	-0.04 [-0.07,-0.02]; P=3e-05
Large HDL (mmol/L)	1.0 (0.4)	0.2 [0.2,0.2]; P=4e-53	-0.06 [-0.09,-0.02]; P=8e-06
Medium HDL (mmol/L)	0.9 (0.3)	0.2 [0.2,0.2]; P=3e-146	-0.02 [-0.04,0.00]; P=0.005
Small HDL (mmol/L)	1.1 (0.2)	0.1 [0.1,0.1]; P=4e-110	0.005 [-0.008,0.018]; P=0.7
**Lipoprotein particle size**			
VLDL particle size (nm)	36 (1)	0.2 [0.1,0.3]; P=2e-06	-0.03 [-0.13,0.07]; P=0.6
LDL particle size (nm)	24 (0)	0.02 [0.01,0.03]; P=0.003	0.0008 [-0.0145,0.0160]; P=0.9
HDL particle size (nm)	10 (0)	0.07 [0.06,0.09]; P=1e-19	-0.06 [-0.08,-0.04]; P=2e-07
**Apolipoproteins**			
Apolipoprotein A-I (g/L)	1.7 (0.2)	0.2 [0.2,0.2]; P=2e-104	-0.06 [-0.08,-0.03]; P=7e-09
Apolipoprotein B (g/L)	0.9 (0.2)	0.03 [0.01,0.04]; P=3e-05	-0.02 [-0.04,0.00]; P=0.07
**Lipids**			
Triglycerides (mmol/L)	1.0 (0.5)	0.2 [0.2,0.2]; P=2e-48	-0.04 [-0.08,0.00]; P=0.09
Phosphoglycerides (mmol/L)	0.9 (0.2)	0.2 [0.2,0.2]; P=1e-225	-0.02 [-0.04,-0.01]; P=0.0003
Cholines (mmol/L)	2.1 (0.5)	0.5 [0.4,0.5]; P=8e-211	-0.05 [-0.09,-0.01]; P=0.0009
Sphingomyelin (mmol/L)	0.3 (0.1)	0.04 [0.03,0.04]; P=6e-53	-0.01 [-0.02,0.00]; P=0.0003
**Cholesterol**			
Total C (mmol/L)	5.1 (1.0)	0.4 [0.3,0.5]; P=1e-31	-0.1 [-0.2,0.0]; P=0.01
Remnant C (mmol/L)	1.4 (0.4)	0.1 [0.1,0.1]; P=1e-17	-0.03 [-0.06,0.01]; P=0.2

VLDL C (mmol/L)	0.7 (0.2)	0.08 [0.06,0.09]; P=9e-28	-0.01 [-0.03,0.01]; P=0.5
IDL C (mmol/L)	0.7 (0.2)	0.03 [0.01,0.04]; P=9e-06	-0.02 [-0.03,0.00]; P=0.1
LDL C (mmol/L)	1.9 (0.6)	0.02 [-0.02,0.06]; P=0.3	-0.03 [-0.08,0.02]; P=0.3
HDL C (mmol/L)	1.8 (0.4)	0.3 [0.2,0.3]; P=3e-86	-0.05 [-0.09,-0.02]; P=6e-05
HDL2 C (mmol/L)	1.2 (0.4)	0.2 [0.2,0.3]; P=1e-71	-0.04 [-0.08,-0.01]; P=0.0004
HDL3 C (mmol/L)	0.5 (0.0)	0.01 [0.01,0.02]; P=1e-12	-0.008 [-0.012,-0.004]; P=6e-05
Esterified C (mmol/L)	3.8 (0.8)	0.3 [0.2,0.3]; P=1e-30	-0.08 [-0.15,-0.01]; P=0.01
**Fatty acids**			
Total FA (mmol/L)	11 (3)	2.2 [2.0,2.4]; P=6e-143	-0.3 [-0.5,-0.1]; P=0.008
Omega-3 FA (mmol/L)	0.4 (0.1)	0.07 [0.06,0.08]; P=4e-60	-0.02 [-0.04,-0.01]; P=3e-05
Docosahexaenoic acid (mmol/L)	0.2 (0.1)	0.06 [0.06,0.06]; P=3e-143	-0.009 [-0.015,-0.003]; P=0.001
Omega-6 FA (mmol/L)	3.8 (0.8)	0.5 [0.4,0.5]; P=6e-80	-0.08 [-0.15,-0.01]; P=0.008
Linoleic acid (mmol/L)	3.2 (0.7)	0.3 [0.3,0.4]; P=2e-47	-0.07 [-0.13,-0.01]; P=0.01
PUFA (mmol/L)	4.2 (0.9)	0.6 [0.5,0.6]; P=4e-87	-0.1 [-0.2,0.0]; P=0.003
MUFA (mmol/L)	3.1 (1.0)	0.7 [0.7,0.8]; P=2e-128	-0.06 [-0.14,0.03]; P=0.2
Saturated FA (mmol/L)	3.6 (1.0)	0.9 [0.8,1.0]; P=2e-158	-0.1 [-0.2,0.0]; P=0.003
**Fatty acid ratios**			
Omega-3 FA (%)	3.7 (1.0)	-0.03 [-0.09,0.04]; P=0.3	-0.1 [-0.2,0.0]; P=0.003
Docosahexaenoic acid (%)	1.7 (0.5)	0.2 [0.2,0.2]; P=1e-31	-0.05 [-0.10,0.00]; P=0.1
Omega-6 FA (%)	35 (4)	-2.2 [-2.4,-1.9]; P=2e-71	0.07 [-0.25,0.39]; P=0.6
Linoleic acid (%)	30 (4)	-2.6 [-2.9,-2.4]; P=1e-95	0.08 [-0.26,0.42]; P=0.6
PUFA (%)	39 (4)	-2.2 [-2.4,-2.0]; P=5e-73	-0.05 [-0.37,0.27]; P=0.9
MUFA (%)	28 (3)	0.9 [0.7,1.2]; P=7e-16	0.2 [-0.1,0.6]; P=0.1
Saturated FA (%)	33 (3)	1.3 [1.1,1.4]; P=1e-49	-0.2 [-0.4,0.0]; P=0.04
Degree of unsaturation	1.3 (0.1)	-0.03 [-0.03,-0.02]; P=4e-21	-0.007 [-0.014,0.000]; P=0.08
**Amino acids**			
Alanine (µmol/L)	423 (69)	16 [11,21]; P=3e-13	-8.0 [-14.0,-2.1]; P=0.009
Glutamine (µmol/L)	497 (81)	-51 [-57,-46]; P=5e-90	-3.0 [-9.8,3.8]; P=0.7
Glycine (µmol/L)	330 (79)	-62 [-67,-57]; P=2e-159	-14 [-20,-7]; P=0.0002
Histidine (µmol/L)	68 (12)	8.1 [7.4,8.9]; P=1e-95	1.5 [0.5,2.5]; P=0.004
Branched-chain amino acids			
Isoleucine (µmol/L)	50 (14)	1.9 [1.0,2.8]; P=7e-08	-0.7 [-1.9,0.6]; P=0.5
Leucine (µmol/L)	80 (17)	5.0 [3.9,6.1]; P=3e-22	0.3 [-1.3,1.8]; P=0.6
Valine (µmol/L)	197 (41)	-5.0 [-7.8,-2.2]; P=0.001	2.3 [-1.4,6.1]; P=0.2
Aromatic amino acids			

Phenylalanine (µmol/L)	81 (13)	9.8 [8.9,10.6]; P=7e-107	-0.4 [-1.6,0.8]; P=0.4
Tyrosine (µmol/L)	51 (12)	-5.3 [-6.1,-4.5]; P=6e-45	-2.2 [-3.3,-1.1]; P=7e-05
**Glycolysis related**			
Glucose (mmol/L)	4.6 (0.7)	-0.06 [-0.10,-0.02]; P=0.001	-0.03 [-0.08,0.03]; P=0.3
Lactate (mmol/L)	1.4 (0.4)	0.09 [0.06,0.11]; P=4e-14	0.02 [-0.01,0.05]; P=0.5
Pyruvate (µmol/L)	83 (25)	4.8 [3.2,6.4]; P=6e-11	-0.09 [-2.14,1.96]; P=0.8
Citrate (µmol/L)	112 (20)	2.5 [1.1,3.8]; P=8e-05	-0.2 [-2.0,1.5]; P=0.8
Glycerol (µmol/L)	105 (43)	6.8 [4.1,9.6]; P=1e-06	-1.7 [-5.6,2.1]; P=0.3
**Ketone bodies**			
Acetoacetate (µmol/L)	60 (40)	2.0 [-0.6,4.6]; P=0.01	4.8 [1.2,8.3]; P=0.005
Beta-hydroxybutyrate (µmol/L)	170 (116)	5.5 [-1.3,12.3]; P=0.06	13 [3,22]; P=0.001
**Miscellaneous**			
Creatinine (µmol/L)	56 (10)	3.6 [3.0,4.3]; P=1e-26	0.9 [0.0,1.8]; P=0.1
Albumin (cu)	0.1 (0.0)	-0.001 [-0.002,-0.001]; P=6e-06	0.0003 [-0.0005,0.0011]; P=0.5
Acetate (µmol/L)	46 (13)	-1.0 [-1.9,-0.2]; P=0.01	-0.3 [-1.5,0.8]; P=0.4
**Hormone related**			
Insulin (mU/L)	7.1 (4.1)	0.6 [0.4,0.9]; P=8e-12	0.2 [-0.2,0.5]; P=0.5
Leptin (ng/mL)	16 (11)	-0.5 [-1.6,0.5]; P=0.8	-0.6 [-1.9,0.7]; P=0.2
Adiponectin (ug/ml)	9.4 (4.3)	1.2 [0.8,1.6]; P=9e-09	-0.3 [-0.8,0.2]; P=0.2
Vitamin D (nmol/L)	24 (11)	2.7 [2.1,3.2]; P=4e-09	1.2 [0.4,1.9]; P=0.003
SHBG (nmol/L)	74 (48)	106 [100,111]; P=2e-241	-8.6 [-16.1,-1.1]; P=2e-05
Testosterone (nmol/L)	1.3 (0.6)	-0.2 [-0.3,-0.2]; P=4e-38	-0.2 [-0.2,-0.1]; P=6e-10
**Inflammation**			
C-reactive protein (mg/L)	2.0 (3.6)	2.1 [1.8,2.3]; P=6e-130	0.2 [-0.2,0.5]; P=0.3
Glycoprotein acetyls (cu)	1.3 (0.2)	0.1 [0.1,0.1]; P=6e-67	-0.02 [-0.04,0.00]; P=0.03

Means and SDs are the average of the means and SDs in each cohort. The cross-sectional associations of 75 molecular measures with the COCP and POC use were meta-analyzed for 5841 women across three independent population-based cohorts. Abbreviations: cu, standardized concentration unit; VLDL, very-low-density lipoprotein; IDL, intermediate-density lipoprotein; LDL, low-density lipoprotein; HDL, high-density lipoprotein; C, cholesterol; FA, fatty acids; PUFA, polyunsaturated fatty acids; MUFA, monounsaturated fatty acids; SHBG, sex hormone-binding globulin. The metabolic measures, except CRP and the six hormone-related measures, were quantified by a high-throughput NMR metabolomics platform.[8] The quality control of this platform has been described previously.[8] Representative CV%s (over thousands of samples) are: Total-C 3.5%, IDL-C 10.3%, remnant-C 12.4%, LDL-C 5.4%, HDL-C 2.7%, total TG 1.7%, IDL-TG 3.2%, LDL-TG 4.4%, LA 4.4%, FAw6 5.2%, FAw6/totFA 2.2%, apoA-I 2.1%, apoB 5.4%, glucose 1.8%, the amino acids and lipoprotein subclass measures are typically around 5%, most of them <5%.

Table S3. Cytokine differences associated with the use of combined oral contraceptive pills and progestin-only contraceptives in absolute concentration units.

Cytokines*	Mean (SD)	Absolute difference associated with COCP use in the cross-sectional setting<br><br>Beta [95%CI]; P-value	Absolute difference associated with POC use in the cross-sectional setting<br><br>Beta [95%CI]; P-value
**Growth factors**			
HGF	438 (168)	-8.4 [-22.7,6.0]; P=0.8	-4.9 [-19.5,9.8]; P=0.9
bNGF	1.5 (0.8)	0.3 [0.2,0.4]; P=3e-05	-0.03 [-0.14,0.09]; P=0.8
SCF	96 (28)	6.5 [3.5,9.5]; P=6e-05	1.0 [-2.0,3.9]; P=0.7
SCGFb	11181 (4597)	3239 [2518,3960]; P=3e-15	197 [-453,846]; P=0.7
G-CSF	144 (73)	11 [5,18]; P=0.04	-5.3 [-11.3,0.7]; P=0.1
VEGF	49 (34)	8.0 [5.2,10.9]; P=9e-11	-0.03 [-2.91,2.85]; P=0.6
FGF basic	51 (28)	7.8 [4.7,10.8]; P=1e-05	-1.9 [-4.9,1.0]; P=0.01
PDGF-BB	5056 (2086)	318 [182,454]; P=3e-09	83 [-58,224]; P=0.3
**Chemokines**			
MCP-1	34 (13)	2.1 [-0.1,4.3]; P=0.01	-1.6 [-3.6,0.4]; P=0.2
MIP-1a	13 (4)	0.9 [0.1,1.6]; P=0.02	-0.6 [-1.3,0.0]; P=0.01
MIP-1b	70 (27)	3.0 [0.3,5.6]; P=0.03	-0.1 [-2.8,2.6]; P=0.7
Eotaxin	91 (49)	-2.1 [-5.7,1.5]; P=0.1	-3.5 [-7.2,0.2]; P=0.02
CTACK	843 (242)	-15 [-54,25]; P=0.4	13 [-23,48]; P=0.4
GROa	96 (49)	7.0 [-1.0,14.9]; P=0.06	-7.4 [-14.6,-0.3]; P=0.02
MIG	560 (446)	164 [91,237]; P=3e-07	16 [-50,82]; P=0.7
IP-10	709 (427)	70 [-1,140]; P=0.01	-16 [-79,48]; P=0.5
SDF-1a	95 (58)	5.9 [0.7,11.1]; P=0.4	-4.7 [-9.6,0.1]; P=0.08
**Interleukins**			
IL-1b	5.3 (2.1)	0.3 [-0.1,0.6]; P=0.09	-0.4 [-0.7,-0.1]; P=0.009
IL-1ra	298 (378)	-8.0 [-31.3,15.2]; P=0.6	-5.5 [-29.6,18.7]; P=0.3
IL-2	22 (22)	3.0 [-0.6,6.6]; P=0.01	-2.8 [-6.0,0.5]; P=0.005
IL-2ra	81 (40)	16 [9,22]; P=0.0001	-2.2 [-8.2,3.7]; P=1
IL-4	7.9 (2.3)	0.5 [0.3,0.8]; P=0.006	-0.2 [-0.4,0.0]; P=0.2
IL-5	6.4 (2.0)	0.3 [-0.1,0.6]; P=0.1	-0.5 [-0.8,-0.2]; P=0.0002
IL-6	13 (11)	1.0 [0.2,1.9]; P=0.1	-0.4 [-1.3,0.5]; P=0.04
IL-7	22 (12)	2.3 [0.3,4.4]; P=0.004	-0.6 [-2.5,1.2]; P=0.06

IL-8	33 (9)	1.9 [0.4,3.4]; P=0.007	-1.0 [-2.4,0.3]; P=0.05
IL-9	139 (580)	-5.0 [-104.3,94.2]; P=0.3	-44 [-133,46]; P=0.02
IL-10	12 (11)	0.5 [0.1,1.0]; P=0.002	0.01 [-0.42,0.44]; P=0.2
IL-12p70	51 (40)	6.0 [3.1,8.8]; P=3e-05	-0.8 [-3.7,2.1]; P=0.05
IL-13	19 (9)	1.2 [-0.3,2.7]; P=0.07	-1.1 [-2.4,0.2]; P=0.04
IL-16	74 (50)	-9.1 [-17.4,-0.9]; P=0.09	4.0 [-3.4,11.5]; P=0.6
IL-17	179 (90)	20 [11,28]; P=1e-05	-4.1 [-12.7,4.6]; P=0.04
IL-18	141 (61)	6.4 [1.8,11.0]; P=0.08	0.01 [-4.18,4.21]; P=0.7
**Tumor necrosis factors**			
TNFa	59 (52)	1.6 [-7.2,10.4]; P=0.1	-4.4 [-12.4,3.5]; P=0.1
TRAIL	106 (71)	-19 [-27,-12]; P=6e-07	-8.0 [-15.4,-0.6]; P=0.3
**Other cytokines**			
MIF	173 (103)	-15 [-32,2]; P=0.2	15 [-1,30]; P=0.1
IFNg	188 (82)	13 [4,21]; P=0.03	-7.9 [-16.3,0.6]; P=0.009

*The units for all cytokine measures are pg/mL. †Available both in YFS and FINRISK. Means and SDs are the average of the means and SDs in YFS and FINRISK (for the measures available in both cohorts). The cross-sectional associations of 37 cytokines with the COCP and POC use were meta-analyzed for 3100 women in YFS and FINRISK (for the measures available in both cohorts). For those measures not available in FINRISK the associations were calculated in YFS for 1101 women.

CV%s were calculated for 10 (apparently healthy) individual's serum samples from duplicates on two different plates. The intra-assay CV%s were typically less than 10%, for example, HGF 4.9% and IL-6 6.0% and the inter-assay CV%s typically less than 15%, for example, HGF 5.8% and IL-6 7.0%. There was quite a lot of variation in the CV%s for different measures, for example, the intra-assay CV% being as low as 2.6% for FGF basic and the inter-assay CV% being as high as 59.7% for bNGF.

Abbreviations: HGF, hepatocyte growth factor; βNGF, beta nerve growth factor; SCF, stem cell factor; SCGFβ, stem cell growth factor beta; GCSF, granulocyte colony-stimulating factor; VEGF, vascular endothelial growth factor; FGF basic, basic fibroblast growth factor; PDGF-BB, platelet derived growth factor BB; MCP–1, monocyte chemotactic protein-1; MIP–1α, macrophage inflammatory protein-1 alpha; MIP–1β, macrophage inflammatory protein-1 beta; CTACK, cutaneous T cell-attracting chemokine; GROα, growth regulated oncogene–alpha; MIG, monokine induced by interferon-gamma; IP–10, interferon gamma-induced protein 10; SDF1α, stromal cell-derived factor-1 alpha; IL, interleukin; TNFα, tumor necrosis factor-alpha; TRAIL, TNF-related apoptosis inducing ligand; MIF, macrophage migration inhibitory factor; IFNγ, interferon-gamma.

Table S4. Contingency table of contraceptive users at baseline and follow-up in YFS.

Counts		Follow-up (2007)					
		Non-users	COCP*	IUS	Implant	Patch†	Vaginal ring†
Baseline (2001)	Non-users	392	52	86	2	0	8
	COCP	94	89	29	1	3	8
	Progestin-only pills	4	2	0	0	0	0
	IUS	32	5	58	1	0	0
	Implant	1	1	0	1	0	0

*At baseline information was available to distinguish COCPs from progestin-only pills but at follow-up these data were not available. However, given that the vast majority of women who used oral contraception were COCP users (92% across all three studies and 97% in YFS baseline), all oral contraceptive users were treated as COCP users at follow-up. See Supplementary Methods for details.

†Newer contraceptive methods (patches and vaginal rings, n=19) which were only used at follow-up, were not analyzed in this study.

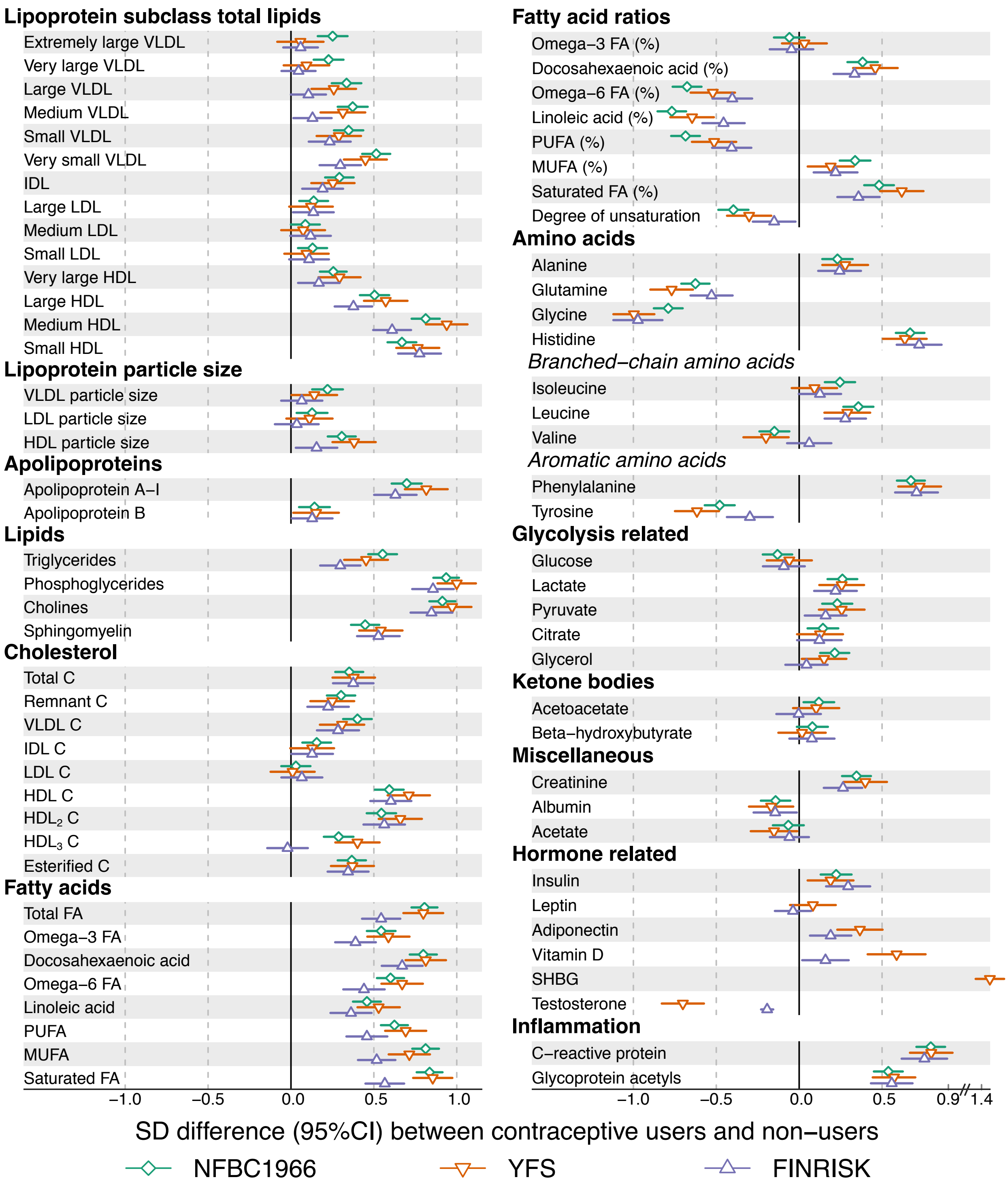

Figure S1. Cross-sectional associations between the use of combined oral contraceptive pills and 75 molecular measures in the individual cohorts. The associations were adjusted for age. Abbreviations are as given in Table S2.

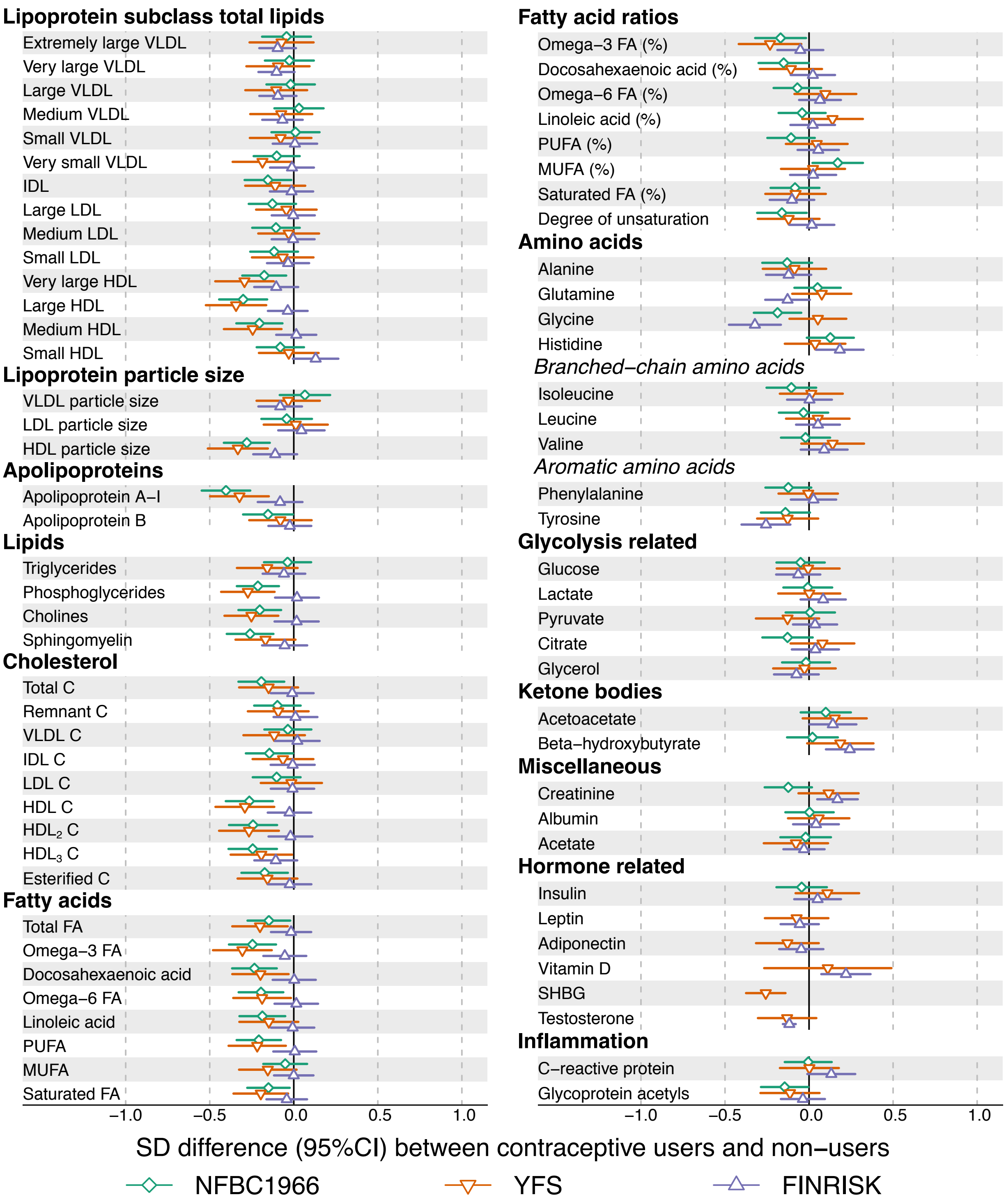

Figure S2. Cross-sectional associations between the use of progestin-only contraceptives and 75 molecular measures in the individual cohorts. The associations were adjusted for age. Abbreviations are as given in Table S2.

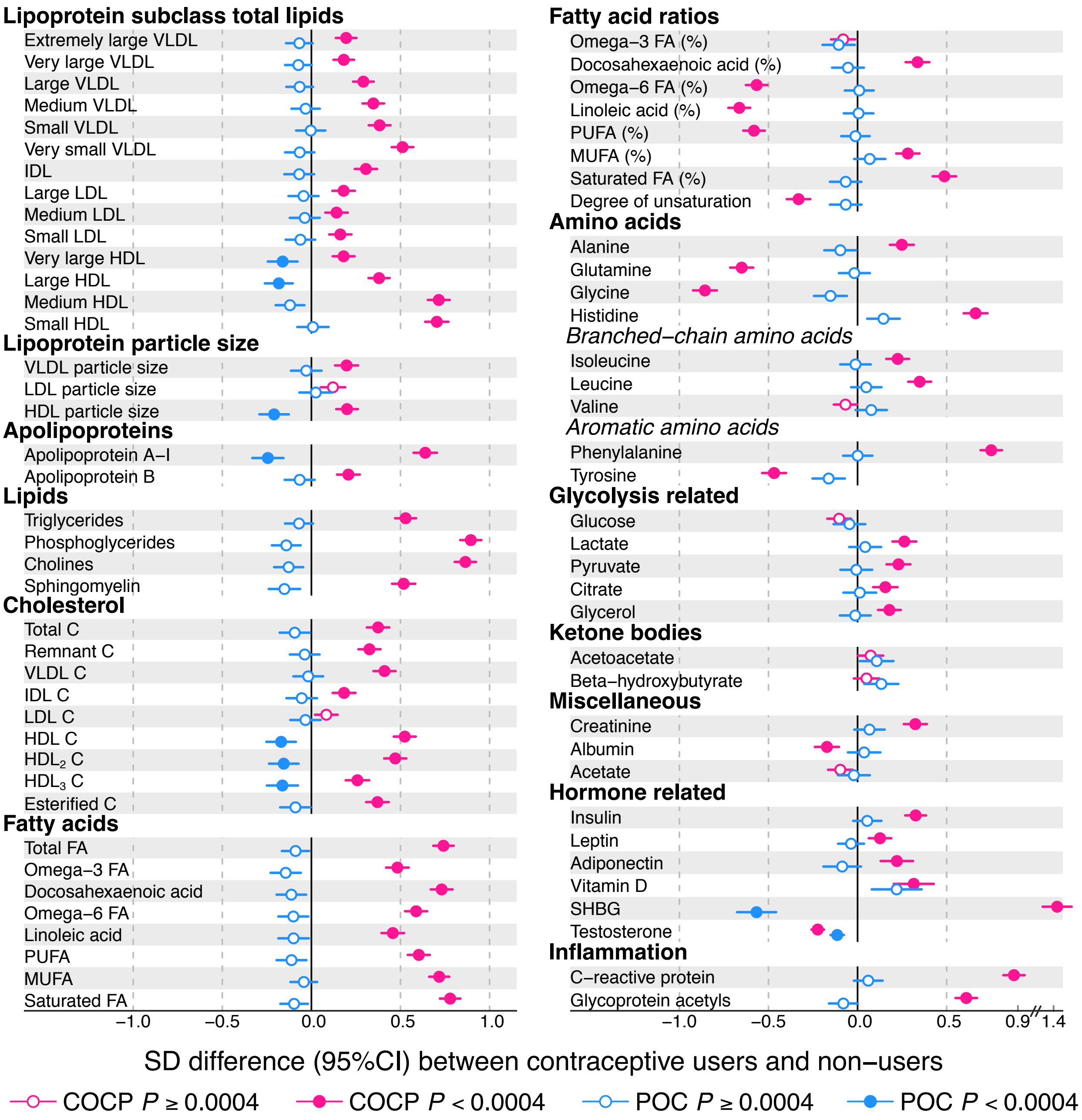

Figure S3. Cross-sectional associations of the use of combined oral contraceptive pills and progestin-only contraceptives with 75 molecular measures, adjusted for age, BMI, mean arterial pressure, smoking and alcohol use. The associations were meta-analyzed via fixed effects model for 5841 women across three independent population-based cohorts. COCP refers to combined oral contraceptive pills and POC to progestin-only contraceptives. Other abbreviations are as given in Table S2.

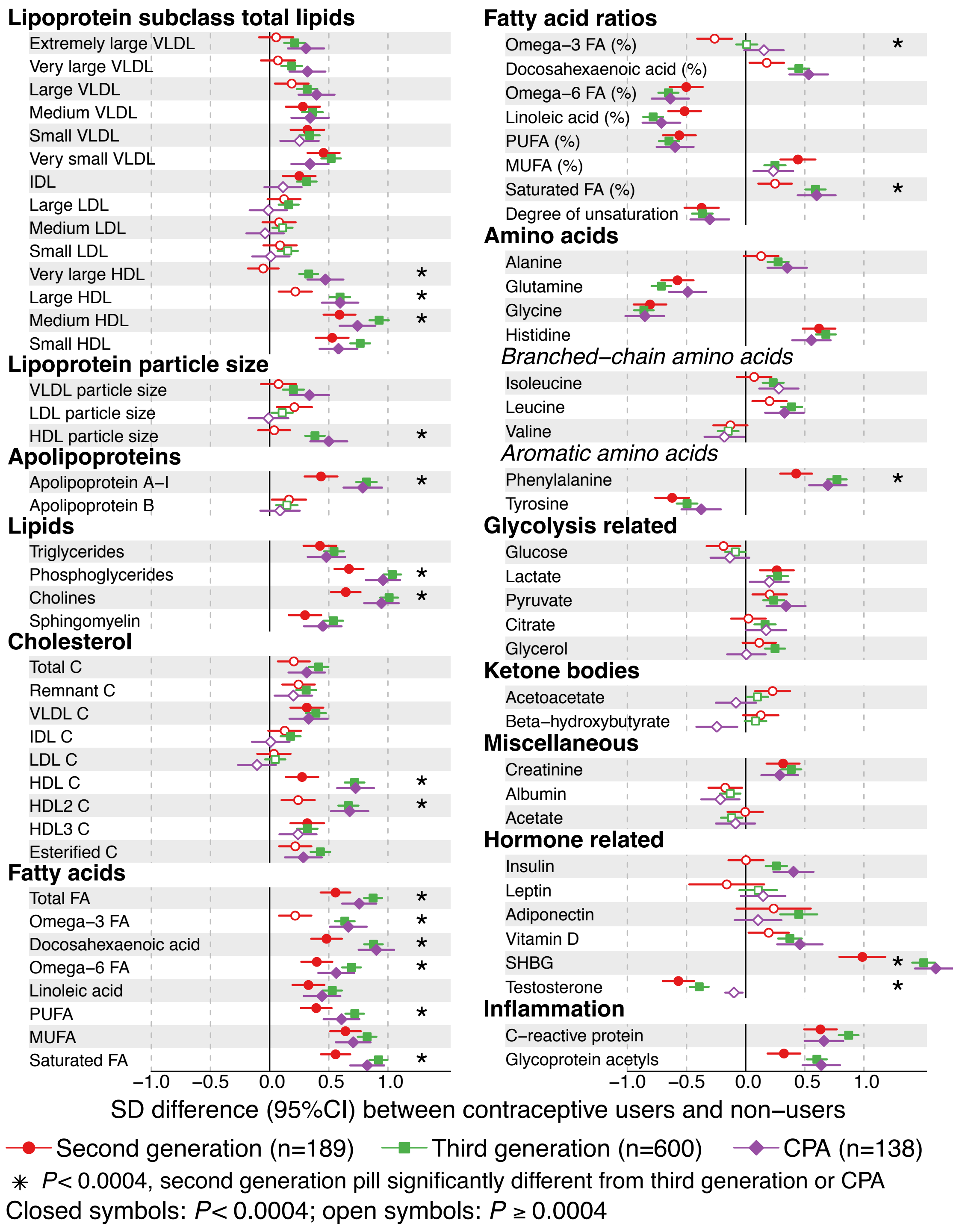

Figure S4. Cross-sectional associations between the use of different subtypes of combined oral contraceptive pills with 75 molecular measures. The associations were adjusted for age, and meta-analyzed via fixed effects model for three independent population-based cohorts. Second generation pills contain estrogen and levonorgestrel and third generation pills estrogen and desogestrel/gestodene. CPA refers to pills containing estrogen and cyproterone acetate. Other abbreviations are as given in Table S2.

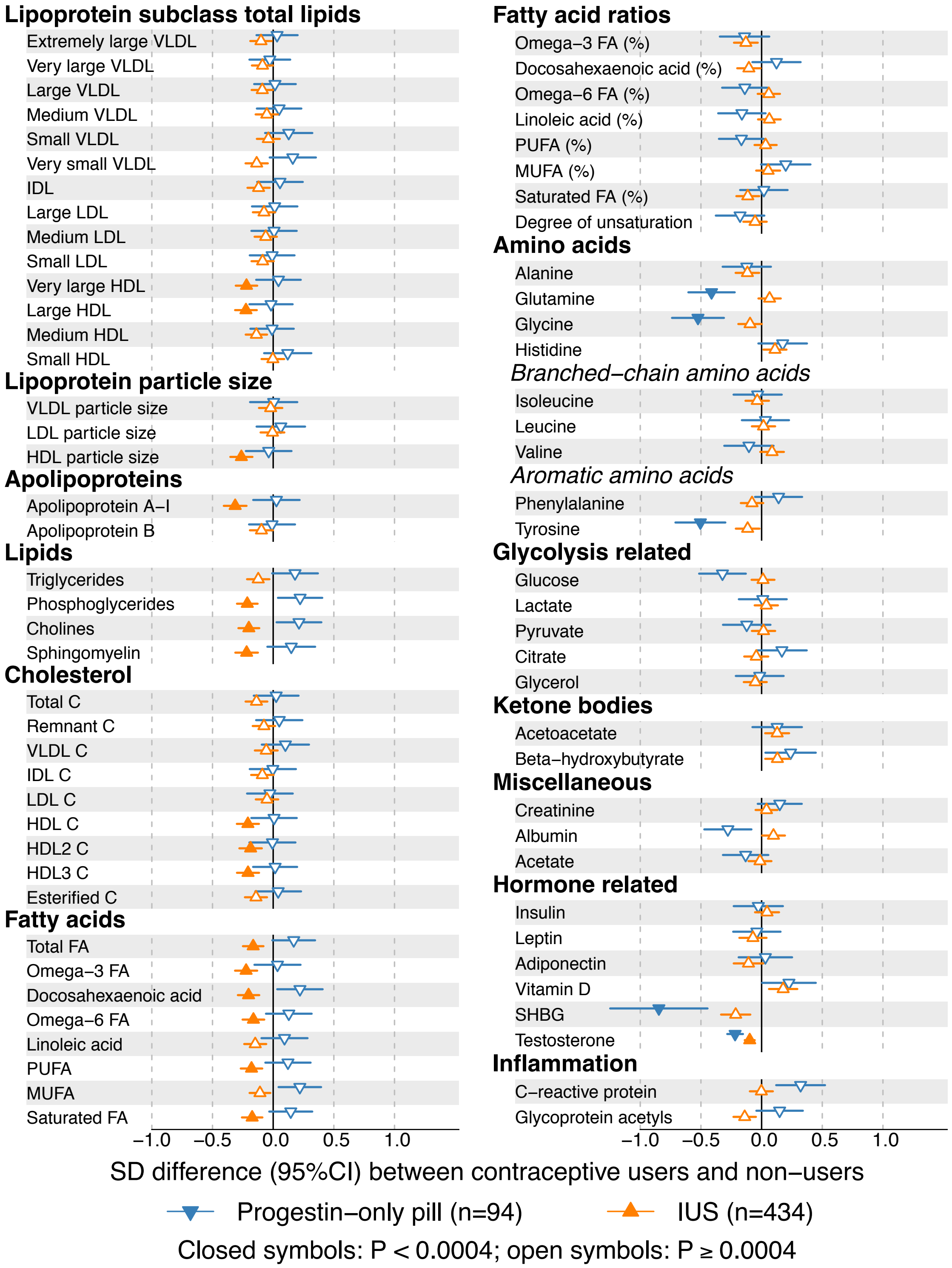

Figure S5. Cross-sectional associations between the use of different subtypes of progestin-only contraceptives with 75 molecular measures. The associations were adjusted for age, and meta-analyzed via fixed effects model for three independent population-based cohorts. Implant users were not included in the analyses since there were only 7 users. IUS refers to intrauterine system. Other abbreviations are as given in TableS2.

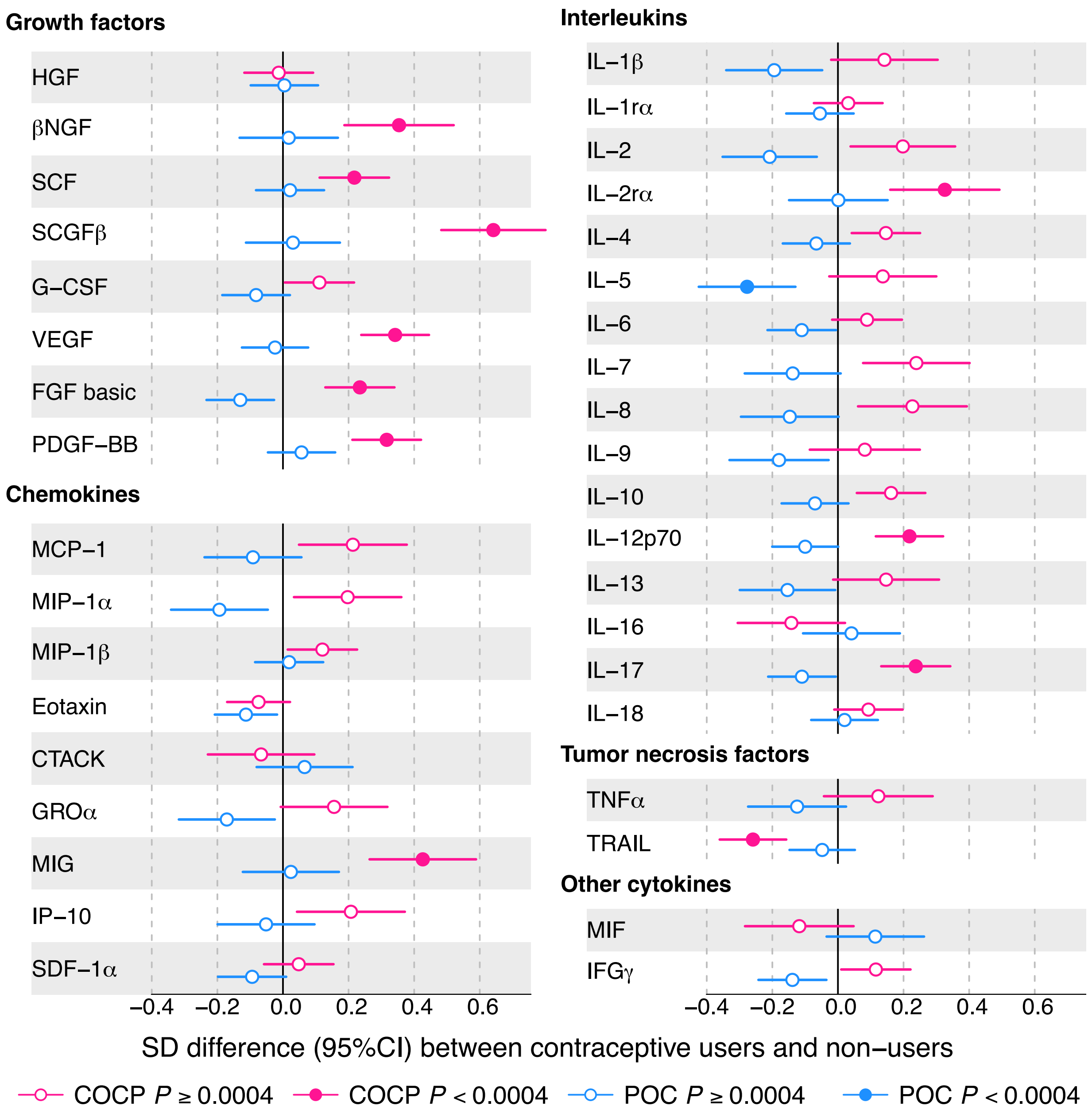

Figure S6. Cross-sectional associations of the use of combined oral contraceptive pills and progestin-only contraceptives with 37 cytokines. Associations were adjusted for age and meta-analyzed for YFS follow-up and FINRISK (when the cytokine measure was available in both cohorts, see S3 Table). In total, 459 women with COCPs and 455 with POCs were compared to 2186 non-users of hormonal contraceptives (when the cytokine measure was available in both cohorts). For those measures only available in YFS, 185 COCP users and 237 POC users were compared to 679 non-users. COCP refers to combined oral contraceptive pills and POC to progestin-only contraceptives. Open and closed circles indicate P≥0.0004 and P<0.0004, respectively. The cytokine abbreviations are as given in Table S3.

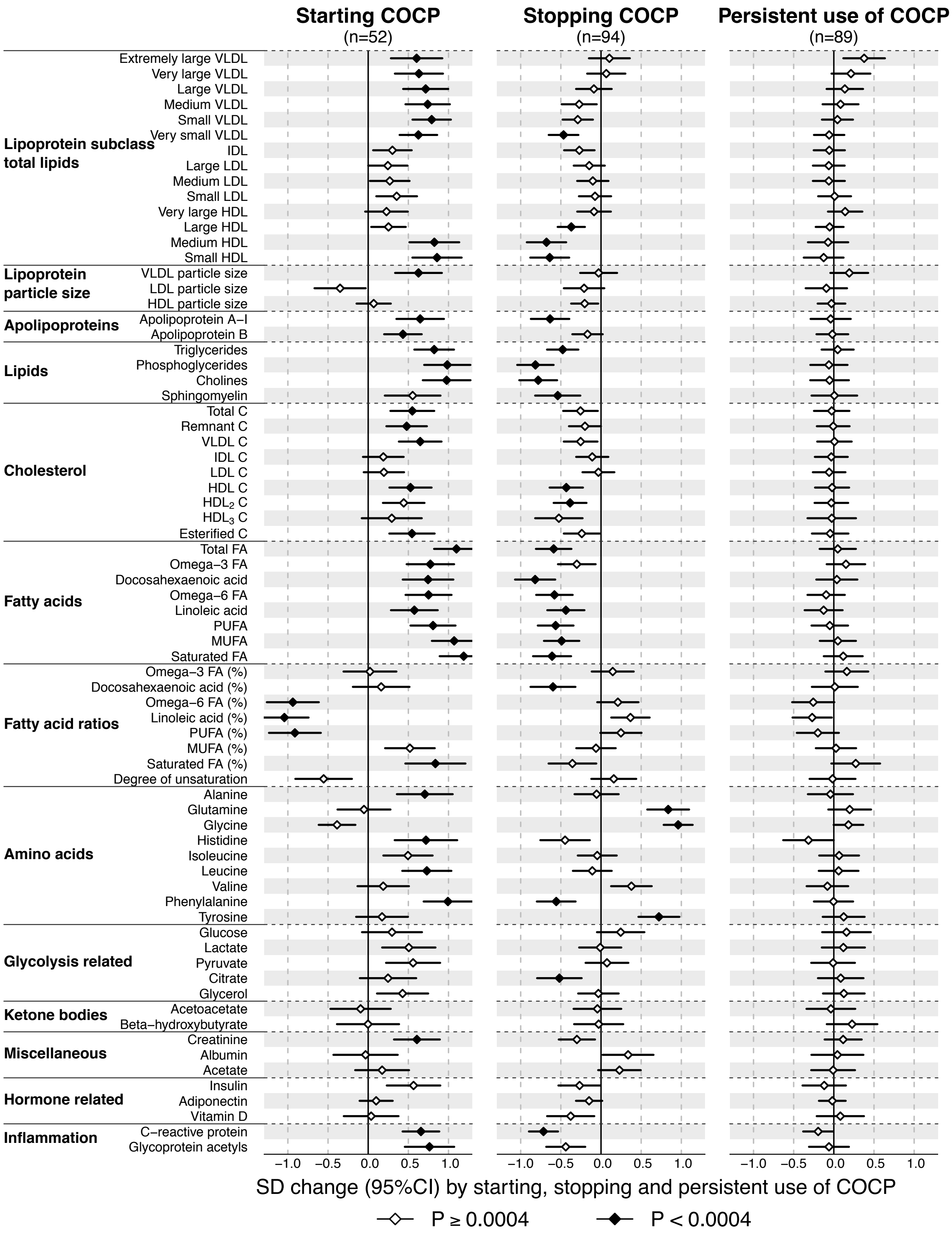

Figure S7. Longitudinal changes in molecular concentrations in response to starting, stopping and persistent use of combined oral contraceptive pills, adjusted for baseline age, and 6-y changes in BMI, mean arterial pressure, smoking and alcohol use. COCP refers to combined oral contraceptive pills and POC to progestin-only contraceptives. Abbreviations are as given in Table S2. Open and closed circles indicate P≥0.0004 and P<0.0004, respectively.

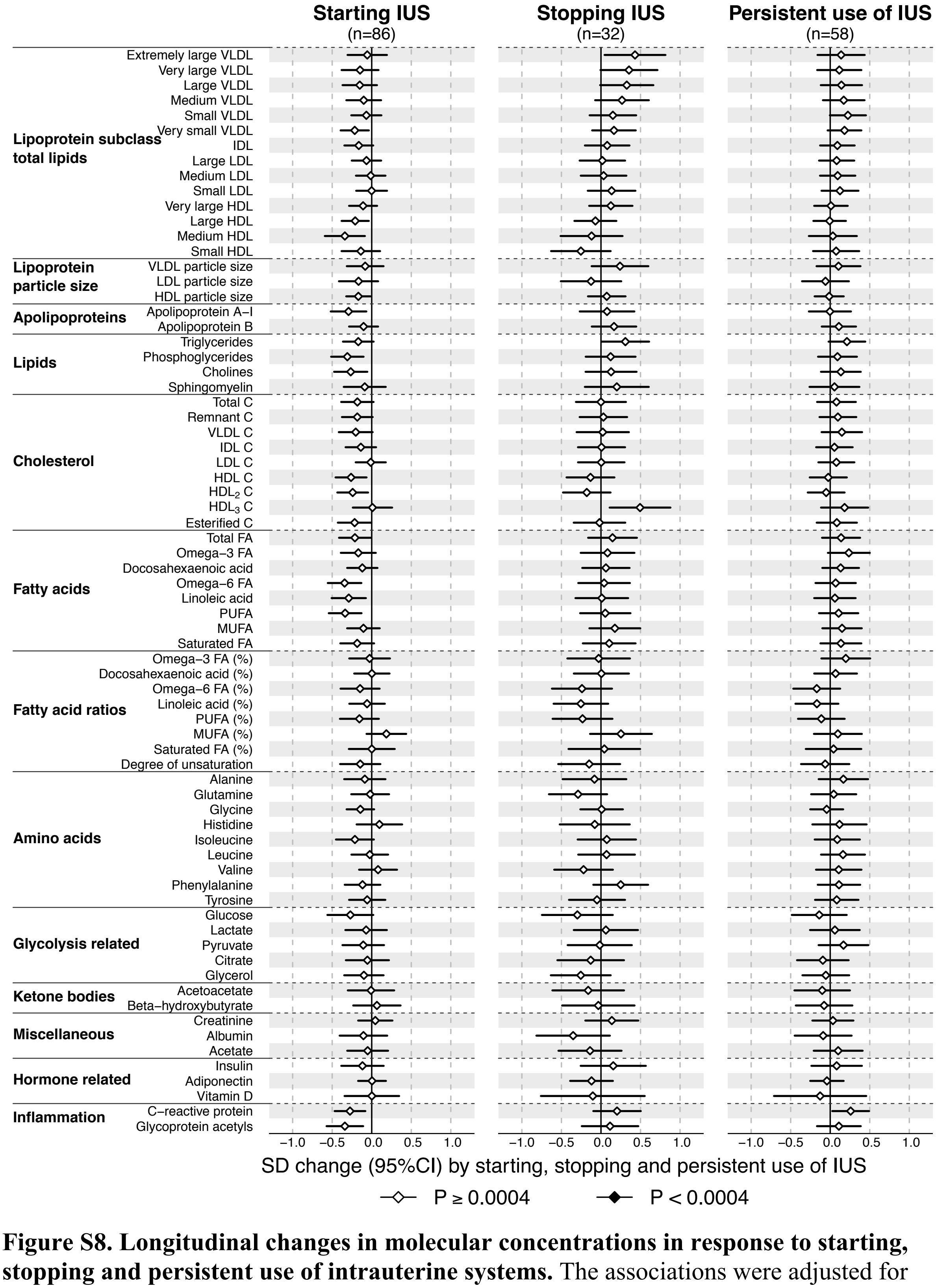

Figure S8. Longitudinal changes in molecular concentrations in response to starting, stopping and persistent use of intrauterine systems. The associations were adjusted for baseline age. IUS refers to intrauterine systems. Other abbreviations are as given in Table S2. Open and closed circles indicate P≥0.0004 and P<0.0004, respectively.

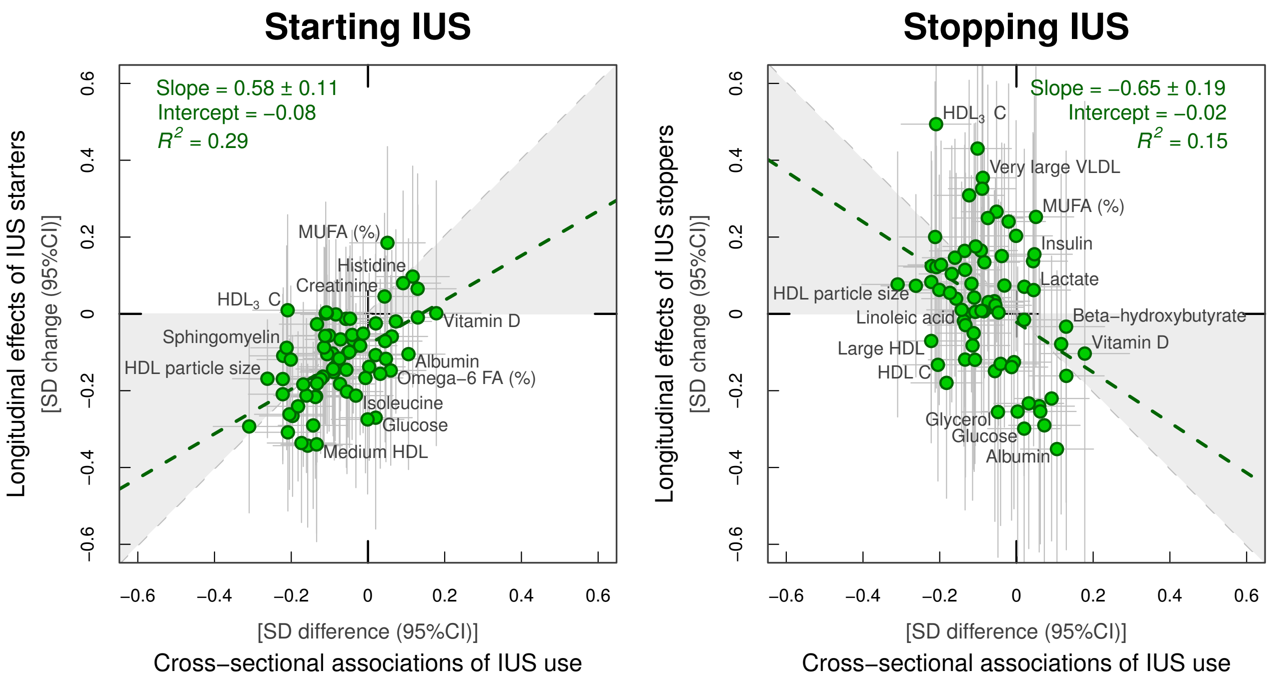

Figure S9. Correlation between cross-sectional and longitudinal metabolic associations with the use of intrauterine systems. The correspondence for starting and stopping the use of IUS is shown on the left and right panel, respectively. Each point represents a metabolic measure. Horizontal and vertical grey lines denote 95% confidence intervals for the cross-sectional and longitudinal associations, respectively. A linear fit of the overall correspondence summarize the match between cross-sectional and longitudinal associations, with R^2 denoting the goodness of fit. A slope of ±1 and R^2=1 would be expected for directly mediated causal effects of IUS on metabolic measures. IUS refers to intrauterine systems. Other abbreviations are as given in Table S2.

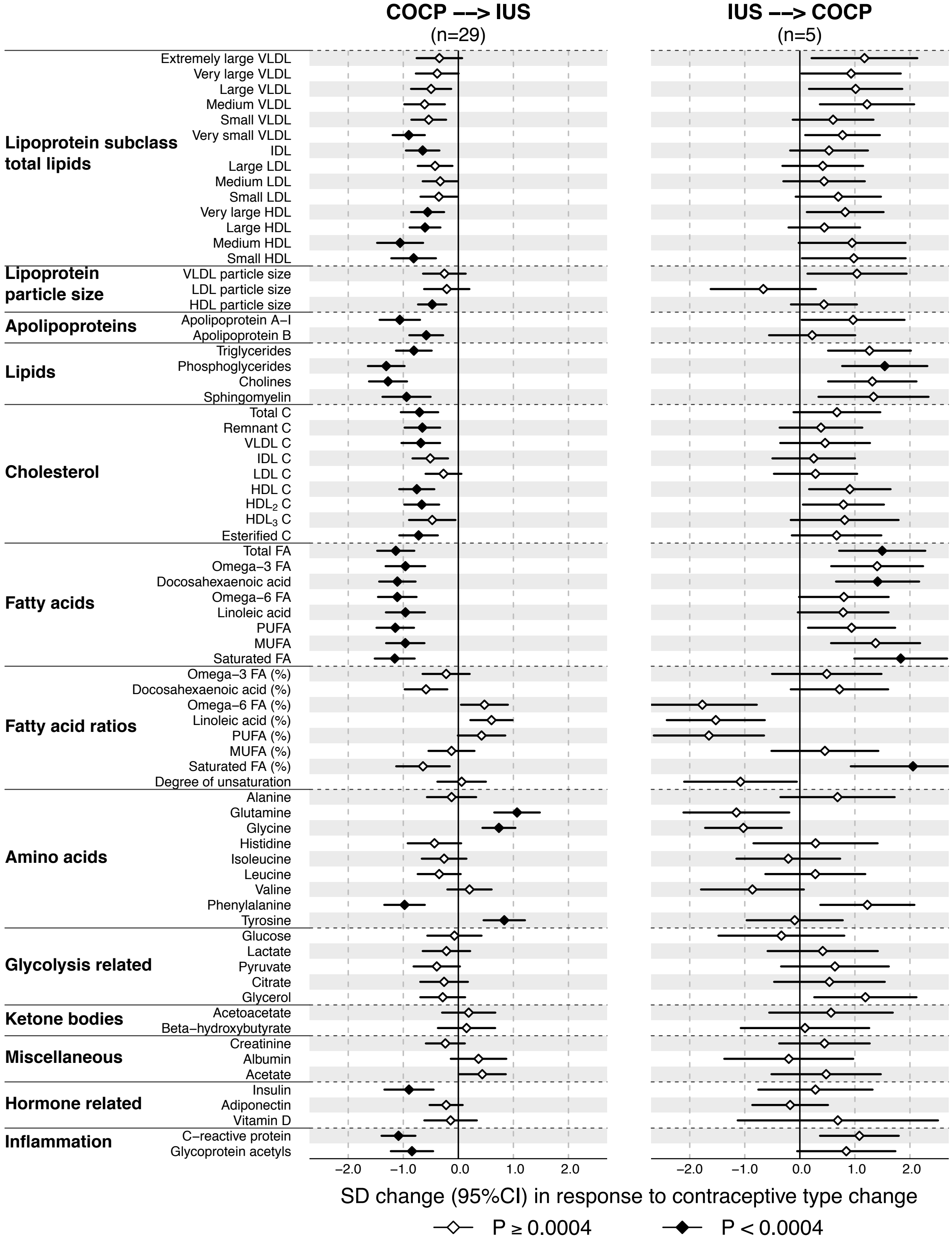

Figure S10. Longitudinal changes in molecular concentrations in response to the change of the contraceptive type. The associations were adjusted for baseline age. The data are from YFS. COCP refers to combined oral contraceptive pills and IUS to intrauterine systems. Abbreviations are as given in Table S2. Open and closed circles indicate P≥0.0004 and P<0.0004, respectively.
